# LNP‐mediated in vivo base editing corrects Agxt to cure primary hyperoxaluria type 1

**DOI:** 10.1002/ctm2.70533

**Published:** 2025-11-23

**Authors:** Dexin Zhang, Rui Zheng, Zhoutong Chen, Xi Chen, Lei Yang, Yanan Huo, Yining Zhao, Jiaxin Huang, Dan Zhang, Shuming Yin, Dali Li, Hongquan Geng

**Affiliations:** ^1^ Department of Urology Children's Hospital of Fudan University Shanghai China; ^2^ Shanghai Frontiers Science Center of Genome Editing and Cell Therapy Shanghai Key Laboratory of Regulatory Biology Institute of Biomedical Sciences and School of Life Sciences East China Normal University Shanghai China; ^3^ Hangzhou Institute of Medicine Chinese Academy of Sciences Hangzhou China; ^4^ Department of Urology Affiliated Yantai Yuhuangding Hospital of Qingdao University Yantai China; ^5^ Shanghai Academy of Natural Sciences (SANS) Shanghai China

**Keywords:** alanine‐glyoxylate aminotransferase gene, base editing, gene editing, lipid nanoparticle, primary hyperoxaluria type 1

## Abstract

**Objective:**

Primary hyperoxaluria type 1 (PH1) is a rare autosomal recessive disorder caused by AGXT mutations, leading to hepatic oxalate overproduction, nephrolithiasis, and progressive renal failure. This study aims to evaluate the therapeutic potential of base editors delivered via lipid nanoparticles (LNPs) for treating PH1.

**Methods:**

We utilized LNPs to deliver the base editor variant spG‐ABE8e into a PH1 rat model. A single‐dose injection of LNP‐ABE was administered to assess its efficacy in correcting the pathogenic Agxt point mutation.

**Results:**

Treatment with LNP‐ABE achieved highly efficient correction of the Agxt mutation, which resulted in the normalization of urinary oxalate excretion, prevention of calcium oxalate deposits, and reversal of renal injury‐associated gene expression profiles in PH1 rats. Furthermore, this study identified the minimum Agxt correction efficiency required for urinary oxalate normalization.

**Conclusion:**

Our findings demonstrate that LNP‐mediated delivery of base editors can effectively correct AGXT pathogenic mutations and ameliorate disease phenotypes in PH1, providing critical preclinical benchmarks for future clinical translation.

**Key points:**

1. The base editor precisely corrected the Agxt gene with high efficiency in PH1 rats.
2. LNP‐delivered Adenine Base Editor (ABE) normalized urinary oxalate levels and prevented calculus formation.
3. This study identified the minimal Agxt correction efficiency required for urinary oxalate normalization.

## Graphical abstract

The base editor precisely corrected the *Agxt* gene with high efficiency in PH1 rats. LNP‐delivered ABE normalised urinary oxalate levels and prevented calculus formation. This study identified the minimal *Agxt* correction efficiency required for urinary oxalate normalisation.

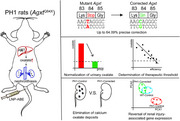

## 1 INTRODUCTION

Primary hyperoxaluria type 1 (PH1) is a rare autosomal recessive metabolic disorder resulting from deficient hepatic alanine‐glyoxylate aminotransferase (AGT) activity, which is caused by mutations in the *AGXT* gene.^1^ AGT catalyses the conversion of glyoxylate to glycine,^2^ preventing excessive glyoxylate oxidation to oxalate. In PH1 patients, overproduced oxalate complexes with calcium to form insoluble calcium oxalate (CaOx) crystals in the kidneys, leading to nephrocalcinosis, recurrent nephrolithiasis, reduced kidney function and ultimately end‐stage renal disease.^3^ PH1 manifests as an early‐onset hereditary disorder, with a median diagnostic age of 5.5 years and approximately 80% of patients achieving diagnostic confirmation before adulthood.^4^, ^5^, ^6^ Of note, 20% of individuals with PH1 are not correctly diagnosed until adulthood^6^ and more frequently present with nephrolithiasis,^7^ underscoring the critical need for targeted clinical management strategies in this patient subgroup.

Current therapeutic approaches for PH1 include supportive therapies (hyperhydration, urine alkalisation and crystallisation inhibitors), pyridoxine supplementation, RNA interference (RNAi) agents, and combined liver and kidney transplantation.^3^, ^8^ Supportive therapies are only capable of delaying disease progression, instead of preventing decline in renal function. Pyridoxine, a cofactor for AGT, can reduce oxalate production by enhancing the activity of AGT protein residues.^9^ While only 30%‒50% of PH1 patients are pyridoxine responsive, relying on the specific *AGXT* genotypes.^6^, ^10^, ^11^ The US Food and Drug Administration has approved two RNAi therapies for PH1: lumasiran^12^ and nedosiran.^13^ Lumasiran targets hepatic glycolate oxidase, while nedosiran inhibits lactate dehydrogenase A, both reducing hepatic oxalate overproduction and consequent urinary oxalate excretion. However, their requirement for lifelong administration leads to high treatment burdens, including substantial costs and adherence challenges, limiting clinical utility. Combined liver‒kidney transplantation remains the only curative intervention to rectify both enzymatic deficiency and organ damage.^3^, ^14^ However, this approach is limited by donor availability and the risks associated with lifelong immunosuppression. The limitations of existing treatments highlight the urgent demand for innovative molecular therapeutic approaches.

Given its pivotal role in oxalate metabolism, the *AGXT* gene is an ideal therapeutic target for developing curative therapies of PH1. Salido et al. demonstrated that adenoviral‐mediated delivery of human *AGXT* cDNA in *Agxt^−/−^* mice significantly restored hepatic AGT activity and decreased urinary oxalate excretion.^15^ Recently, Taihua et al. developed an mRNA‐based protein replacement therapy for PH1 and achieved a 70% reduction in urinary oxalate in PH1 rats.^16^ Nevertheless, longitudinal assessment revealed a time‐dependent diminution of therapeutic efficacy, correlating with a progressive decline in transgene expression. Gene‐editing technology, particularly the CRISPR/Cas9 system, offers a promising approach to achieving long‐term therapeutic efficacy.^17^ Nieto‐Romero et al. used CRISPR/Cas9 to generate site‐specific *AGXT* correction cells from PH1 patients, which could reverse oxalate accumulation in vitro.^18^ However, CRISPR/Cas9‐mediated homologous‐directed repair achieved with this approach is inefficient.^19^ Furthermore, this investigation lacked in vivo experimental validation data.

Base editing technology enables precise and efficient single‐nucleotide conversions, demonstrating the therapeutic potential for rectifying pathogenic single‐nucleotide variants (SNVs) associated with inherent disorders.^20^, ^21^, ^22^, ^23^ In our previous study, AAV‐delivered base editors partially corrected *Agxt* mutations in PH1 rats, yielding measurable but incomplete therapeutic effects.^24^ However, the clinical translation of AAV‐delivered base editors is hindered by constrained packaging capacity, pre‐existing immunity and persistent transgene expression.^24^, ^25^, ^26^, ^27^

Recent advancements in lipid nanoparticles (LNPs) technology have accelerated its application in gene‐editing tool delivery. Compared to AAV, LNPs offer several advantages.^28^ LNPs possess superior cargo capacity, enabling the encapsulation of complete base editor components within individual nanoparticles. LNPs exhibit low immunogenicity, allowing repeated in vivo administration without triggering immune reactions. Furthermore, LNPs eliminate the risk of genomic integration while simultaneously reducing the cytotoxicity and off‐target risks associated with prolonged expression of gene‐editing tools. Therefore, LNPs hold broad prospects for in vivo gene editing in the treatment of diseases.

In our previous work, we developed a *PH1* rat model that carries a nonsense (*Agxt* p.Q84X) mutation that results in a complete lack of AGT protein expression and develops robust hyperoxaluria early in life, closely mirroring the aggressive disease phenotype observed in a significant subset of PH1 patients.^29^ Although the specific mutation studied is not the most common, it provides a clean background for precisely evaluating the efficacy and allows us to unequivocally determine the minimum editing efficiency and the corresponding threshold of AGT protein expression required to achieve a meaningful biochemical and phenotypic correction.

In this study, we employed LNPs to deliver a base editor variant, spG‐ABE8e, to correct the *Agxt* mutation for the treatment of PH1. A single‐dose injection of LNP‐ABE in PH1 rats achieved highly efficient correction of *Agxt* point mutations, restoring hepatic AGT expression to normal levels. Treatment reduced urinary oxalate excretion to physiological levels, eliminated renal CaOx deposition and kidney damage, and reversed the expression of renal injury‐associated genes. Through correlation and linear regression analyses between correction efficiency and therapeutic outcomes, we identified that approximately 44% correction of *Agxt* pathogenic mutations is required to normalise urinary oxalate excretion in PH1 rats. Collectively, our study demonstrates that LNP‐delivered base editors can effectively correct *AGXT* pathogenic mutations for PH1 treatment, providing critical benchmarks for clinical translation.

## 2 RESULTS

### 2.1 Screening for optimal base editors to correct the *Agxt^Q84X^* pathogenic variant in vitro

**FIGURE 1:**
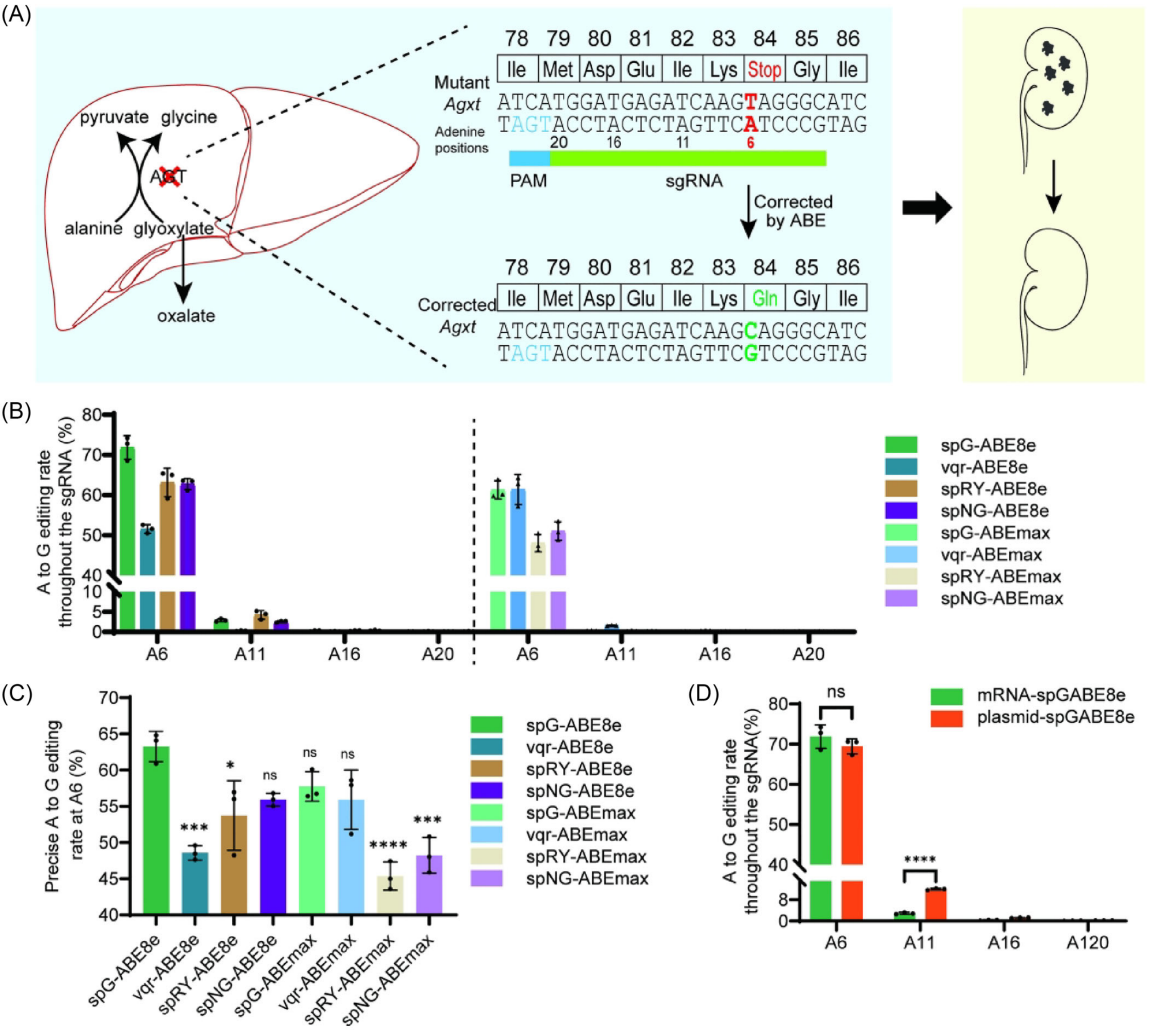
In vitro screen of optimal base editor variants to correct the pathogenic *Agxt* variant. (A) Schematic illustration of *AGXT* pathogenic mutation correction using base editors for primary hyperoxaluria type 1 (PH1) therapy. The targeted adenine (A6) on the sgRNA is highlighted in red, while bystander sites (A11, A16 and A20) are marked in black. (B) Comparison of DNA editing efficiency of all adenine (A) sites within the sgRNA in *Agxt*‐Q84X cell lines 72 h post‐transfection with eight candidate base editor variants (*n* = 3). (C) Evaluation of precise correcting efficiencies at the target site (A6) by different base editor variants (*n* = 3). (D) Comparison of DNA editing efficiency of all adenine (A) sites within the sgRNA in *Agxt*‐Q84X cell lines 72 h post‐transfection with SpG‐ABE8e plasmid or mRNA (*n* = 3). Data are mean (standard deviation [SD]). *p*‐Values were calculated using one‐way analysis of variance (ANOVA). ns, *p *> .05; ^*^ *p* < .05; ^**^ *p *< .005; ^***^ *p* < .0005; ^****^ *p* < .0001.

To screen the optimal base editors systems, we first designed the sgRNA with an “NGA” PAM (N, represents the base A/T/C/G), which places the *Agxt* c.250 T pathogenic variant in protospacer position 6 (A6) and additional adenine bases at protospacer position 11, 16 and 20 (A11, A16 and A20) (Figure 1A), and developed several ABE systems, which could recognise the “NGA” PAM, including SpG‐ABE8e/max, vqr‐ABE8e/max, SpNG‐ABE8e/max and SpRY‐ABE8e/max. Next, *Agxt‐Q84X* cell lines were generated as previously,^24^ and chemically synthesised sgRNA was co‐transfected into *Agxt*‐Q84X cell lines together with these ABEs mRNA. Next‐generation sequencing (NGS) revealed that spG‐ABE8e achieved the highest efficiency (71.87%) at the target site A6 (*Agxt* c.250 T), followed by spRY‐ABE8e (63.17%) and spNG‐ABE8e (62.77%), while vqr‐ABE8e showed reduced activity (Figure 1B). Notably, ABEmax variants displayed significantly lower off‐target editing at bystander sites (A11, A16 and A20) (Figure 1B). Based on in silico predictions (PROVEAN),^30^ we found that the bystander edits at sites A11 (the main bystander site leading *Agxt* p.I82T mutation) would affect AGT protein function (Table S1). Thus, precise correcting efficiency at the target site A6 was analysed, and spG‐ABE8e demonstrated the highest perfect editing rate at A6 (63.26%), followed by spG‐ABEmax (57.75%), spNG‐ABE8e (55.93%) and vqr‐ABEmax (55.91%) (Figure 1C). Therefore, ABE8e‐SpG was selected for subsequent experiments.

To compare DNA‐ and RNA‐mediated base editing systems, we co‐transfected spG‐ABE8e mRNA or plasmid DNA into *Agxt‐Q84X* cell lines. NGS analysis showed comparable editing efficiencies at the target A6 site between the two delivery modalities at 72 h post‐transfection (Figure 1D). Notably, mRNA delivery achieved substantially lower bystander editing rates (2.9% vs. 11.9%; *p *< .0001), yielding a higher precise base editing rate at A6 (68.47% vs. 53.80%; *p *< .0001) (Figure 1D).

### 2.2 Correction of *Agxt^Q84X^* pathogenic variant by LNP‐ABE in PH1 rats

To validate LNPs targeting specificity and transient expression profile, we formulated firefly luciferase (Luc)‐encoding mRNA into LNP and injected LNP‐Luc into 3‐week‐old rats via tail vein. We found that luminescent signals localised predominantly to the upper abdominal region 6 h after injection (Figure S1A), followed by rapid signal attenuation (Figure S1A,B). Ex vivo imaging of dissected organs (heart, kidney, liver, lung and spleen) at 6‐h post‐injection confirmed hepatospecific expression patterns (Figure S1C). These results collectively confirmed that the LNP‐mRNA delivery system achieves targeted hepatic delivery and enables transient transgene expression in vivo.

**FIGURE 2:**
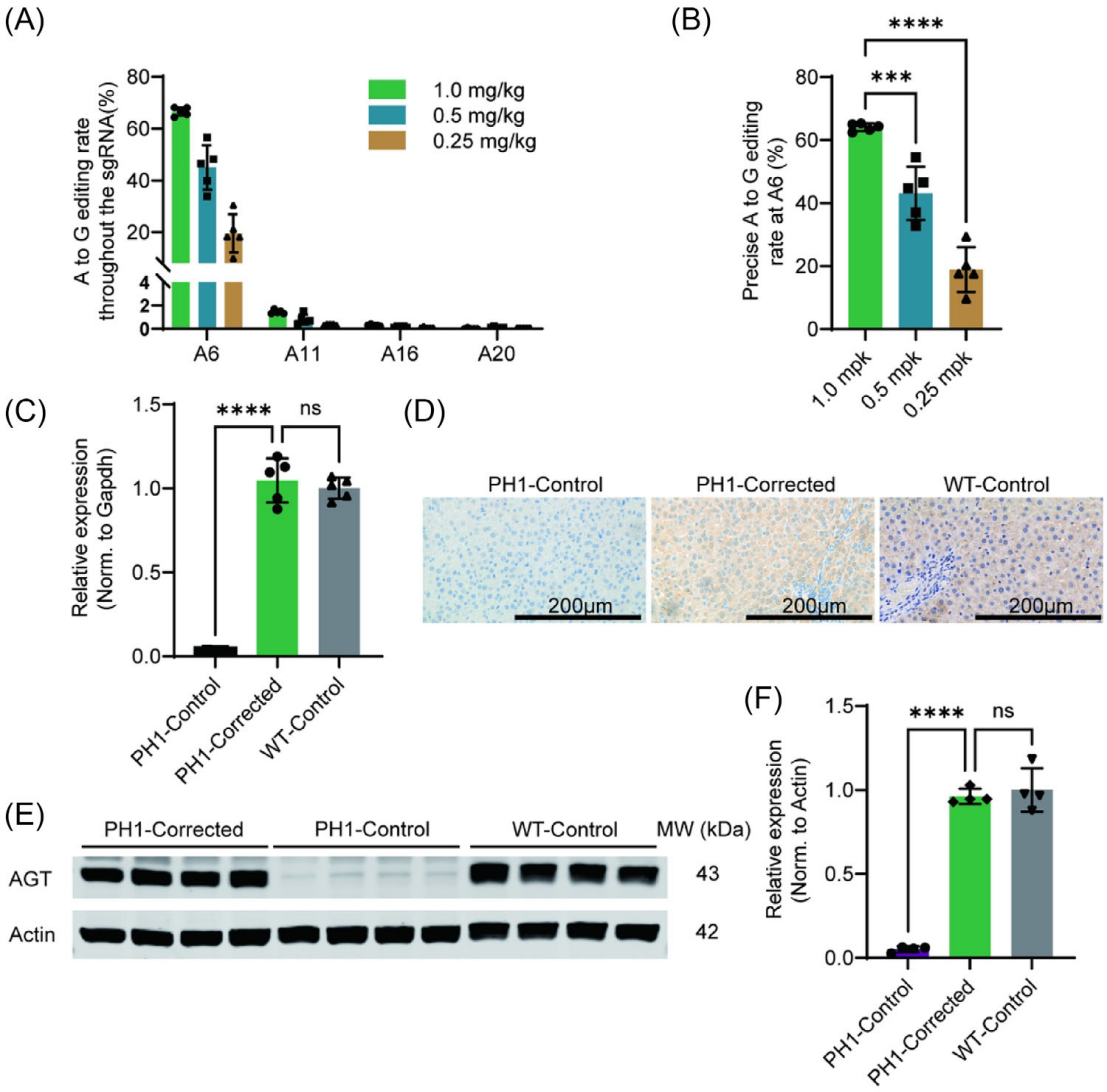
Lipid nanoparticle (LNP)‐ABE‐mediated precise correction of *Agxt* pathogenic variant and normalised alanine‐glyoxylate aminotransferase (AGT) protein expression in primary hyperoxaluria type 1 (PH1) rats. (A) Assessment of DNA editing efficiency of all adenine (A) sites within the sgRNA in vivo 7 days following tail vein injection of different doses of LNP‐ABE (*n* = 5). (B) Precise on‐target editing for different doses of LNP‐ABE (*n* = 5). (C) RT‐qPCR analysis of relative *Agxt* mRNA expression levels in whole liver tissues among three experimental groups (*n* = 5). (D) Immunohistochemical (IHC) staining showing elevated AGT protein expression in the whole liver of three experimental groups. (E) Western blot analysis of AGT protein expression in whole liver tissue of PH1‐Corrected, PH1‐Control and wild‐type (WT)‐Control rats. (F) Quantitative analysis of AGT protein expression levels. The AGT band intensity was normalised to Actin and expressed relative to that of WT‐Control rats. Data are mean (standard deviation [SD]). *p*‐Values were calculated using one‐way analysis of variance (ANOVA). ns, *p *> .05; ^*^ *p* < .05; ^**^ *p *< .005; ^***^ *p* < .0005; ^****^ *p* < .0001.

Next, we tested the optimal dosage in vivo. Three doses (.25,.5 and 1.0 mg/kg) were evaluated for their capacity to correct the target site A6. LNP‐ABE complexes encapsulating spG‐ABE8e mRNA and chemically synthesised sgRNA were administered via tail vein injection to 3‐week‐old PH1 rats. NGS analysis of liver biopsies 7 days post‐injection revealed that the editing efficiencies at both the target site A6 and main bystander editing sites A11 exhibit a dose‐dependent increase (Figures 2A and S2) and precise correcting efficiency at the target site A6 was 18.89% (.25 mg/kg), 43.10% (.5 mg/kg) and 64.09% (1.0 mg/kg) (Figure 2B). Given that hepatocytes constitute 60%‒70% of liver cells, the 1.0 mg/kg dose achieved near‐saturating correction^31^ (PMID: 34012082). Thus, we set PH1 rats receiving 1.0 mg/kg LNP‐ABE as PH1‐Corrected group, with phosphate‐buffered saline (PBS)‐treated PH1 rats (PH1‐Control) and PBS‐treated wild‐type (WT‐Control) rats as the control for the following research. Multi‐parametric analysis (RT‐qPCR [Figure 2C], immunohistochemistry [Figure 2D‒F] and immunoblotting [Figure 2E, F]) demonstrated complete restoration of hepatic *Agxt* expression in PH1‐Corrected group to WT‐Control group levels, with undetectable expression in PH1‐Control livers.

### 2.3 LNP‐ABE modulated hepatic gene expression patterns in PH1 rats

The most immediate consequence of *AGXT* gene mutations is the overproduction of oxalate in the liver. Accumulating evidence has linked *AGXT* dysfunction and primary hyperoxaluria with liver disease,^32^, ^33^ with clinical studies demonstrating significant liver involvement in PH1 patients being more pronounced.^34^ Therefore, we hypothesised that the correction of *Agxt* mutations to reduce hepatic oxalate production could modulate liver transcriptional programs, potentially reversing gene expression profiles associated with metabolic dysfunction and hepatic inflammation.

**FIGURE 3:**
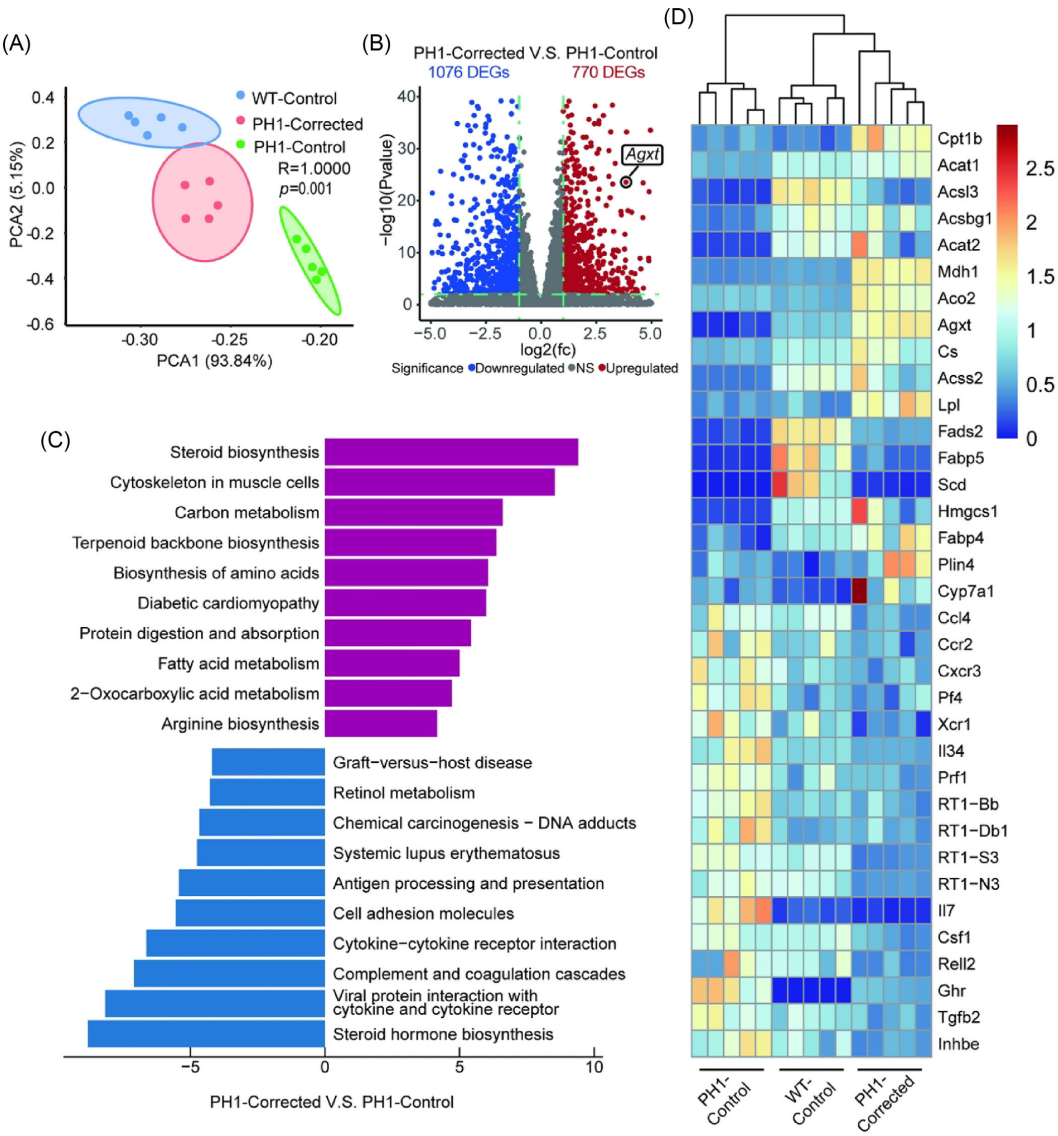
Lipid nanoparticle (LNP)‐ABE modulated hepatic gene expression patterns in primary hyperoxaluria type 1 (PH1) rats. (A) Principal component analysis (PCA) was performed based on RNA sequencing (RNA‐seq) of livers from PH1‐Corrected, PH1‐Control and wild‐type (WT)‐Control rats (*n* = 5). (B) Volcano plot of differentially expressed genes (DEGs) significantly upregulated (red) or downregulated (blue) in livers from PH1‐Corrected and PH1‐Control rats (*n* = 5) based on RNA‐seq (*n* = 5). (C) Top 10 enriched pathways of the upregulated DEGs (red) and downregulated DEGs (blue) based on Kyoto Encyclopedia of Genes and Genomes (KEGG) pathway analysis comparing livers from PH1‐Corrected and PH1‐Control rats (*n* = 5). (D) Heatmap of DEGs related to liver function and injury comparing livers from PH1‐Corrected, PH1‐Control and WT‐Control rats (*n* = 5).

To evaluate the hepatic molecular responses to LNP‐ABE therapy, we performed bulk RNA sequencing (RNA‐seq) of livers from PH1‐Corrected, PH1‐Control and WT‐Control rats at 7 days after injection. Principal component analysis revealed that the gene expression pattern of livers from PH1‐Corrected rats was distinct from PH1‐Control rats, importantly, clustered more closely with that of the WT‐Control group (Figure 3A). Volcano plot revealed over 1800 differentially expressed genes (DEGs), with 1076 significantly reduced and 770 significantly elevated in livers from PH1‐Corrected rats compared with PH1‐Control rats (Figure 3B). Kyoto Encyclopedia of Genes and Genomes (KEGG) pathway analysis revealed pronounced enrichment of upregulated genes in core hepatic metabolic pathways, particularly steroid biosynthesis, carbon metabolism and fatty acid β‐oxidation, critical to hepatocellular homeostasis. Conversely, downregulated genes exhibited robust clustering within immune‐inflammatory activation pathways (Figure 3C). RNA‐seq analysis (Figure 3D) revealed that key genes related to fatty acid degradation (including *Cpt1b*, *Acat1*, *Acsl3*, *Acsbg1*), the PPAR signalling pathway (including *Lpl*, *Fads2*, *Scd*, *Hmgcs1* and *Fabp4*), and glyoxylate and dicarboxylate metabolism (including *Agxt*, *Mdh1* and *Aco2*) were significantly upregulated in livers from PH1‐Corrected rats. Importantly, the expression levels of these genes in the PH1‐Corrected group were restored to levels more comparable to those in the WT‐Control group. Moreover, proinflammatory factors, chemokines and antigen‐presenting molecules were significantly decreased in the livers of PH1‐Corrected rats. These results demonstrate that our therapeutic intervention not only corrected the *Agxt* gene expression in PH1 rat livers but also positively influenced liver‐related gene expression.

### 2.4 Safety assessment of LNP‐ABE‐treated PH1 rats

**FIGURE 4:**
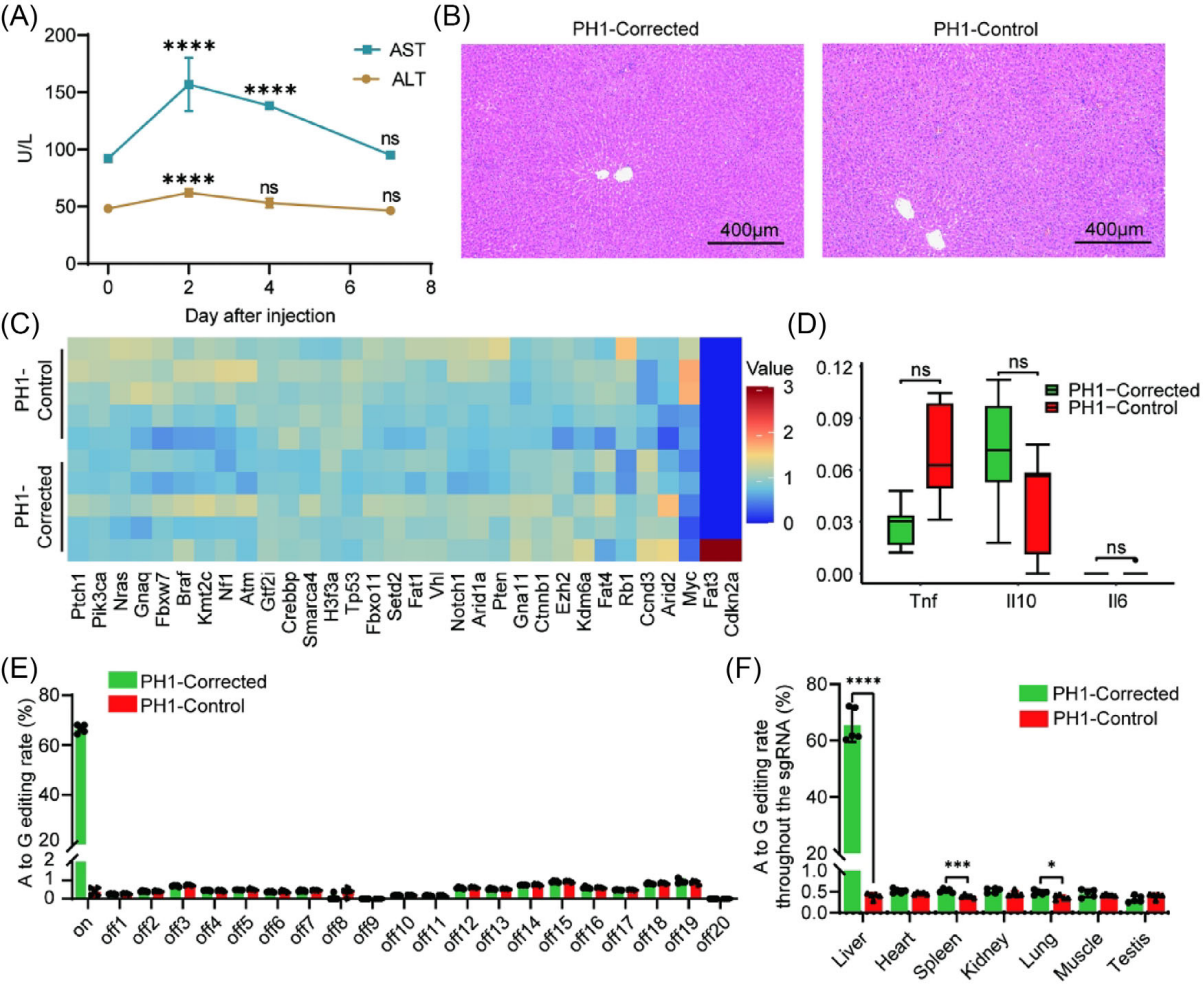
Assessment of hepatic toxicity and off‐target editing in primary hyperoxaluria type 1 (PH1) rats. (A) Changes in aspartate aminotransferase (AST) level and alanine aminotransferase (ALT) level in PH1‐Corrected rats at various time points following treatment with 1 mg/kg dose of lipid nanoparticle (LNP)‐ABE (*n* = 5). (B) Representative haematoxylin‒eosin (H&E)‐stained liver tissues from PH1‐Corrected and PH1‐Control rats 1 week after injection. (C) Bulk RNA sequencing (RNA‐seq) heatmap illustrating similar transcriptional profiles of cancer‐associated genes in the liver biopsy samples taken 7 days after treatment of PH1‐Corrected and PH1‐Control rats. (D) Bulk RNA‐seq boxplot showing the expression of *Tnf*, *Il10* and *Il6* gene in the liver biopsy samples taken 7 days after treatment of PH1‐Corrected and PH1‐Control rats (*n* = 5). (E) Cumulative A‐to‐G editing within the protospacer region of the top 20 predicted off‐target loci in the liver tissue from 6‐month‐old PH1‐Corrected and PH1‐Control rats (*n* = 5). (F) A‐to‐G editing rates of the targeted site (A6) in various mouse organs of 6‐month‐old PH1‐Corrected and PH1‐Control rats (*n* = 5). Data are mean (standard deviation [SD]). *p*‐Values were calculated using unpaired Student's *t*‐tests. ns, *p *> .05; ^*^ *p* < .05; ^**^ *p *< .005; ^***^ *p* < .0005; ^****^ *p* < .0001.

We evaluated hepatic toxicity through monitoring changes in serum alanine aminotransferase (ALT) and aspartate aminotransferase (AST) levels, histological assessment and analysis of liver injury‐associated gene expression. Serum ALT and AST levels exhibited transient elevation, peaking at 48 h post‐administration and then gradually returning to baseline levels (Figure 4A). This result aligned with previous studies.^35^, ^36^ Histological analysis via haematoxylin‒eosin (H&E) staining revealed no evidence of hepatocellular necrosis, inflammatory infiltration or architectural distortion in liver sections obtained 1 week post‐treatment (Figure 4B), confirming the absence of clinically significant hepatic damage despite transient transaminase elevation. Bulk RNA‐seq of liver tissue demonstrated that LNP‐ABE administration did not induce significant upregulation of cancer and inflammatory related genes (Figure 4C, D).

To assess potential off‐target effects, we utilised CRISPR RGEN Tools^37^ to predict the top 20 candidate off‐target sites (Table S2) and analysed these sites by NGS. NGS analysis revealed no detectable off‐target editing activity at these sites in hepatic tissue (Figure 4E). Furthermore, we performed whole‐genome sequencing (WGS) on PH1‐Corrected and PH1‐Control rats. The WGS analysis did not identify significant off‐target editing above background levels, supporting the high specificity of our therapeutic strategy (Figure S3A). To investigate extrahepatic editing, multiple organs (heart, liver, spleen, kidneys, lungs, skeletal muscle and testes) were harvested from PH1‐Corrected and PH1‐Control rats. NGS analysis identified low yet statistically significant editing of the *Agxt^Q84X^* pathogenic variant in in spleens and lungs of PH1‐Corrected rats, with other tissues displaying background signal levels (Figure 4F). Since the *Agxt* is a liver‐specific expression gene^38^ and our treatment strategy is correcting *Agxt* pathogenic variant, these minimal extrahepatic edits are considered unlikely to possess toxicological significance.

To address the potential for systemic immune responses or inflammation triggered by LNP‐ABE, we measured the expression levels of key immune and inflammatory markers—including interleukin (IL)‐6, IL‐1β, IL‐10 and CD68—in multiple tissues (heart, spleen, lung, skeletal muscle and testis). Our results showed no significant differences in the expression of these markers between the LNP‐ABE‐treated group and the control group, suggesting that the treatment did not elicit a notable systemic immune or inflammatory response under the experimental conditions tested (Figure S3B‒F).

### 2.5 LNP‐ABE eliminates the PH1 phenotypes in rats

**FIGURE 5:**
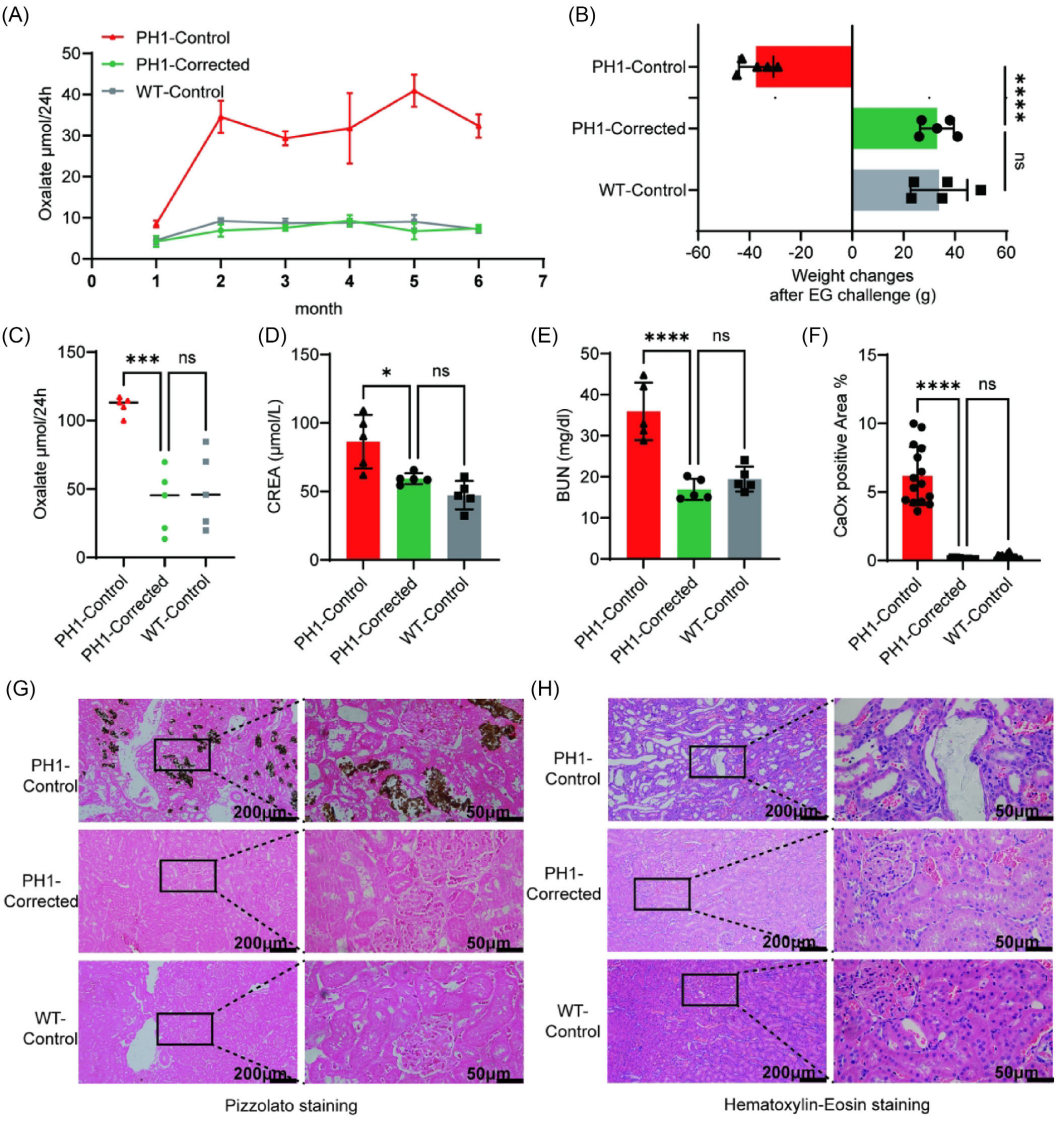
Lipid nanoparticle (LNP)‐ABE reliably rescues the disease phenotype in primary hyperoxaluria type 1 (PH1) rats. (A) Time course of urinary oxalate levels changes in wild‐type (WT)‐Control, PH1‐Control and PH1‐Corrected rats (*n* = 5). (B) Body weight changes in WT‐Control, PH1‐Control and PH1‐Corrected rats during the 10‐day ethylene glycol (EG) challenge (*n* = 5). (C) Measurement of urinary oxalate levels in WT‐Control, PH1‐Control and PH1‐Corrected rats on day 10 of the EG challenge (*n* = 5). (D) Serum creatinine (CREA) levels in WT‐Control, PH1‐Control and PH1‐Corrected rats after the 10‐day EG challenge (*n* = 5). (E) Serum blood urea nitrogen (BUN) levels in WT‐Control, PH1‐Control and PH1‐Corrected rats after 10‐day EG challenge (*n* = 5). (F) Quantification of CaOx deposition areas in renal tissues of WT‐Control, PH1‐Control and PH1‐Corrected rats after the 10‐day EG challenge. The ratio of CaOx deposits was quantified in three randomly selected fields per renal tissue section (*n* = 5). (G) Representative Pizzolato staining of renal sections from WT‐Control, PH1‐Control and PH1‐Corrected rats after the 10‐day EG challenge. (H) Representative haematoxylin‒eosin (H&E) staining of renal sections from WT‐Control, PH1‐Control and PH1‐Corrected rats after the 10‐day EG challenge. Data are mean (standard deviation [SD]). *p*‐Values were calculated using one‐way analysis of variance (ANOVA). ns, *p *> .05; ^*^ *p* < .05; ^**^ *p *< .005; ^***^ *p* < .0005; ^****^ *p* < .0001.

Since PH1 rats develop hyperoxaluric beginning at 1 month of age, we monitored 24‐h urinary oxalate excretion across all three experimental groups from 1 to 6 months of age. PH1‐Corrected rats exhibited substantially decreased urinary oxalate levels compared to PH1‐Control rats (49.8%, 81.4%, 71.9%, 69.7%, 83.6% and 78.3% mean reduction at monthly intervals from 1 to 6 months), and were indistinguishable from WT‐Control rats (Figure 5A and Table S3). This indicates that LNP‐ABE‐mediated correction of *Agxt* successfully renormalised oxalate biosynthesis.

To further evaluate therapeutic efficacy, we analysed 24‐h urinary oxalate levels and renal CaOx deposition in all three groups following the 10‐day ethylene glycol (EG) challenge. Body weight measurements obtained pre‐ and post‐challenge revealed comparable weight gain patterns in PH1‐Corrected and WT‐Control groups, whereas PH1‐Control rats showed significant weight loss (Figure 5B). The urine oxalate levels on day 10 of the EG challenge showed elevated levels across all groups. Notably, oxalate excretion in PH1‐Corrected rats remained comparable to WT‐Control rats and significantly lower than PH1‐Control rats (Figure 5C).

After the EG challenge, rats were euthanised and kidneys and serum were collected. Serum biochemistry revealed significantly improved renal function in PH1‐Corrected rats, with creatinine (CREA) (Figure 5D) and blood urea nitrogen (BUN) (Figure 5E) levels comparable to WT control rats and marked improvement over PH1‐Control rats. Histopathological assessment via Pizzolato staining revealed minimal CaOx crystal deposition in the kidney of PH1‐Corrected and WT‐Control rats (Figure 5G), contrasting with extensive CaOx deposition (6.17% renal area occupancy) in PH1‐Control rats (Figures 5F and S4). H&E staining confirmed that *Agxt* mutation correction reversed characteristic PH1 histopathology including tubular dilatation, epithelial sloughing, cast formation and crystalline deposition (Figure 5H), achieving full phenotypic rescue.

### 2.6 *Agxt* mutation correction rescues renal pathological gene expression in PH1 rat

**FIGURE 6:**
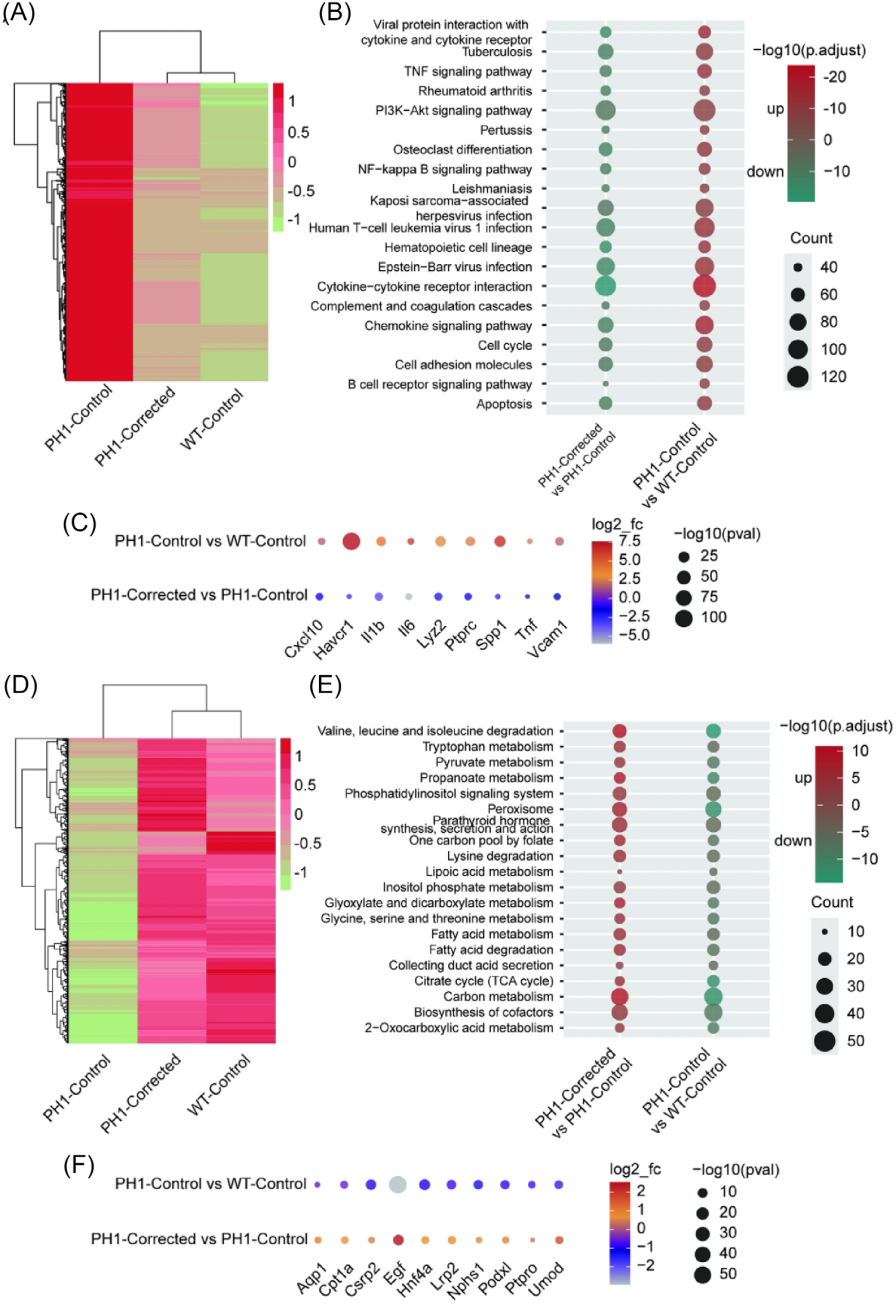
Efficient correction of *Agxt* pathogenic mutations reverses abnormal gene expression in kidneys of primary hyperoxaluria type 1 (PH1) rats. (A) Heatmap shows the number of differentially expressed genes (DEGs) that were upregulated in PH1‐Control rats compared with wild‐type (WT)‐Control rats but downregulated by lipid nanoparticle (LNP)‐ABE treatment (up‒down pattern) (*n* = 5). (B) Dot plot of Kyoto Encyclopedia of Genes and Genomes (KEGG) pathway analysis in DEGs that followed the up‒down pattern. (C) Dot plot shows the representative DEGs that follow the up‒down pattern in different groups. (D) Heatmap shows the number of DEGs that were downregulated in PH1‐Control rats compared with WT‐Control rats but upregulated by LNP‐ABE treatment (down‒up pattern) (*n* = 5). (E) Dot plot of KEGG pathway analysis in DEGs that followed the down‒up pattern. (F) Dot plot shows the representative DEGs that follow the down‒up pattern in different groups.

Studies indicate that nephrolithiasis induces renal injury and modulates renal gene expression programs.^39^ To investigate the impact of the correction of *Agxt* mutations in renal gene expression profiles in PH1 rats, bulk RNA‐seq was performed on renal tissues following the EG challenge. RNA‐seq transcriptomic analysis revealed 2924 genes exhibiting significant upregulation in PH1‐Control rats versus WT‐Control rats were effectively re‐downregulated in the PH1‐Corrected rats (Figure 6A). Gene Ontology (GO) term and pathway analysis of these re‐downregulated genes revealed enrichment for functions highly relevant to renal pathogenesis: including viral protein‐cytokine/cytokine receptor interaction, Tumor Necrosis Factor (TNF) signalling, Phosphoinositide 3‐kinase Protein Kinase B (PI3K‒Akt) signalling, Nuclear Factor Kappa‐Light‐Chain. Enhancer of Activated B cells (NF‐κB) signalling, cytokine‒cytokine receptor interaction, complement and coagulation cascades, chemokine signalling and cell adhesion molecule pathways (Figure 6B). Key upregulated genes in PH1‐Control kidneys, including chemokine‐related *Cxcl10* and *Il1b*, cytokine‐associated *Il6* and *Tnf*, renal injury markers *Havcr1* and *Vcam1*, immune cell regulators *Ptprc* and *Lyz2*, and fibrotic marker *Spp1*, demonstrating pronounced inflammatory responses, immune cell activation, tubular epithelial damage and fibrotic progression (Figure 6C). These transcriptional aberrations were ameliorated following therapeutic intervention.

Conversely, 2424 genes downregulated in the kidneys of PH1‐Control rats relative to WT‐Control rats exhibited restored expression levels post‐treatment (Figure 6D). The correction of *Agxt* pathogenic mutations reversed these transcriptional abnormalities, rescuing the expression of genes essential for renal homeostasis. Significant disruption was observed in key metabolic pathways, including the citrate cycle (Tricarboxylic Acid (TCA) cycle), fatty acid metabolism and pyruvate metabolism, in PH1‐Control rats. In contrast, PH1‐Corrected rats demonstrated marked upregulation of these pathways relative to PH1‐Control rats (Figure 6E). Suppressed functional markers in PH1‐Control rats included *Aqp1*, *Lrp2* and *Umod* (impaired tubular water/electrolyte/protein reabsorption); *Nphs1*, *Podxl* and *Ptpro* (glomerular filtration barrier dysfunction); *Egf* and *Csrp2* (compromised tubular repair/regenerative capacity); and *Cpt1a* and *Hnf4a* (disrupted energy metabolism and functional regulation), indicating severe renal impairment (Figure 6F).

In summary, *Agxt* mutation correction not only restored transcriptional networks regulating inflammatory responses, immune activation and renal injury but also ameliorated structural abnormalities, metabolic dysregulation and functional impairment.

### 2.7 Correction of *Agxt* pathogenic variant and rescue of PH1 phenotypes in adult rats

The accurate diagnosis of patients with PH1 is frequently delayed due to insufficient clinical awareness and limited access to genetic testing.^6^ This specific patient population, differing from those with early‐onset disease, experiences prolonged exposure to PH1‐associated pathologies including hyperoxaluria before initiating targeted therapies. The PH1 mouse model develops hyperoxaluria exclusively during adulthood, thus failing to accurately recapitulate the disease progression observed in this patient population.^15^ The PH1 rat model used in this study develops disease manifestations during juvenile stages with established hyperoxaluria before reaching adulthood, thereby providing a suitable preclinical platform for evaluating therapeutic efficacy in adult PH1 cases.

**FIGURE 7:**
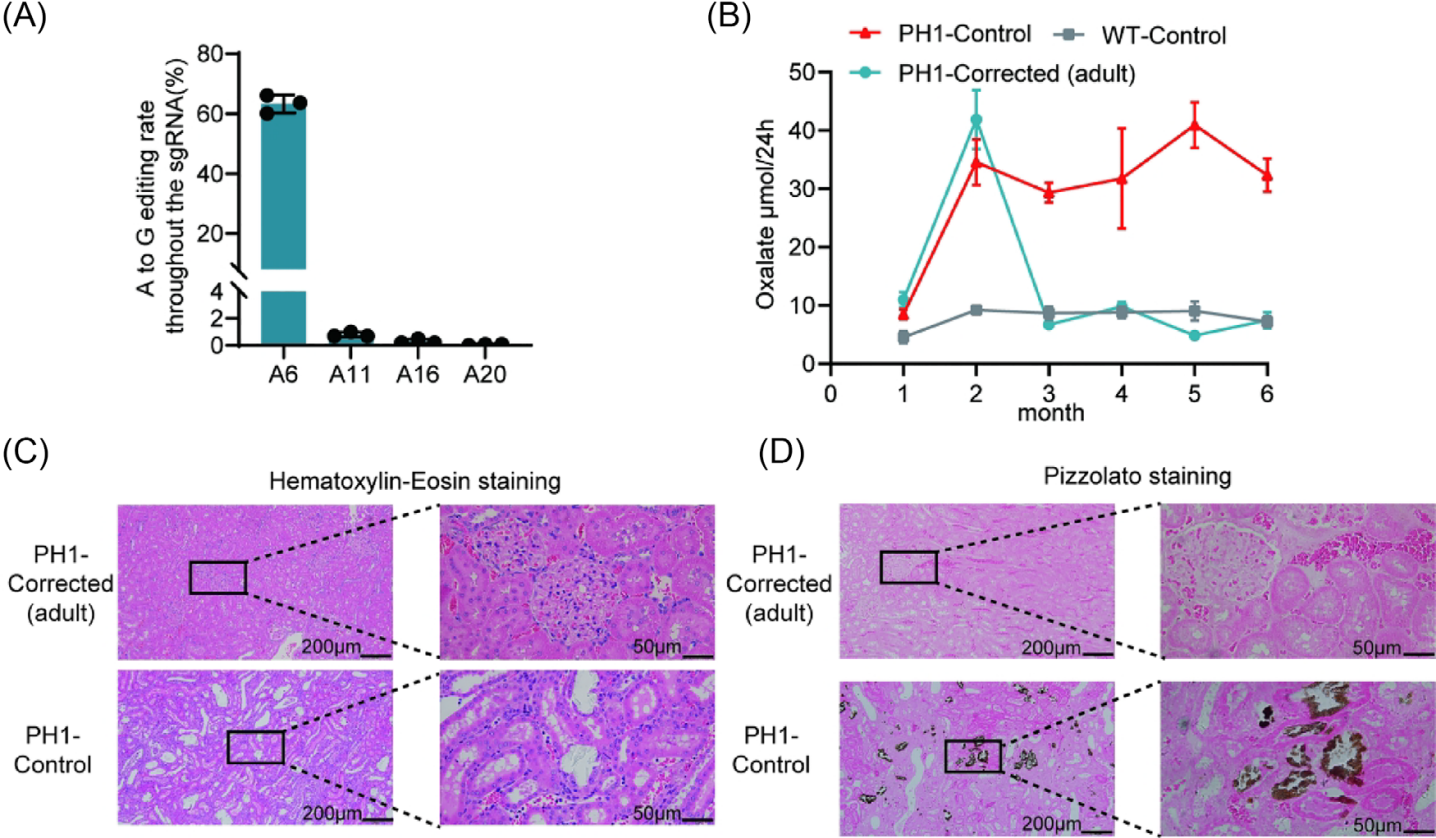
Lipid nanoparticle (LNP)‐ABE‐mediated precise correction of the *Agxt* pathogenic variant and rescued the disease phenotype in adult primary hyperoxaluria type 1 (PH1) rats. (A) Assessment of DNA editing efficiency of all adenine (A) sites within the sgRNA in liver tissues of 6‐month‐old PH1‐Corrected (adult) rats (*n* = 3). (B) Time course of urinary oxalate levels changes in PH1‐Corrected (adult) rats post‐LNP‐ABE administration. (C) Representative haematoxylin‒eosin (H&E) staining of renal sections from PH1‐Corrected (adult) and PH1‐Control rats after the 10‐day ethylene glycol (EG) challenge. (D) Representative Pizzolato staining of renal sections from PH1‐Corrected (adult) and PH1‐Control rats after the 10‐day EG challenge. Data are mean (standard deviation [SD]).

To assess the therapeutic efficacy of LNP‐mediated base editor mRNA delivery for correcting *Agxt* pathogenic mutations in adults, we administered 1.0 mg/kg LNP‐mRNA via tail vein injection to 2‐month‐old PH1 model rats. NGS analysis of liver tissue genomic DNA demonstrated efficient hepatic editing following adult‐stage administration at 1.0 mg/kg (Figures 7A and S5). Twenty‐four‐hour urinary oxalate excretion was initially elevated in pre‐treatment PH1 rats compared to WT controls, normalised after treatment and remained stable through 6 months of age (Figure 7B). Histopathological evaluation via H&E and Pizzolato staining of renal tissue after EG challenge, adult‐treated PH1 rats showed absence of significant pathological alterations (Figure 7C) and CaOx deposition (Figure 7D). These findings demonstrate that LNP‐mRNA administration during adulthood enables efficient correction of *Agxt* pathogenic mutations and achieves phenotypic reversal of PH1 rats.

### 2.8 Therapeutic threshold assessment of *Agxt* pathogenic variant correction for PH1 treatment

**FIGURE 8:**
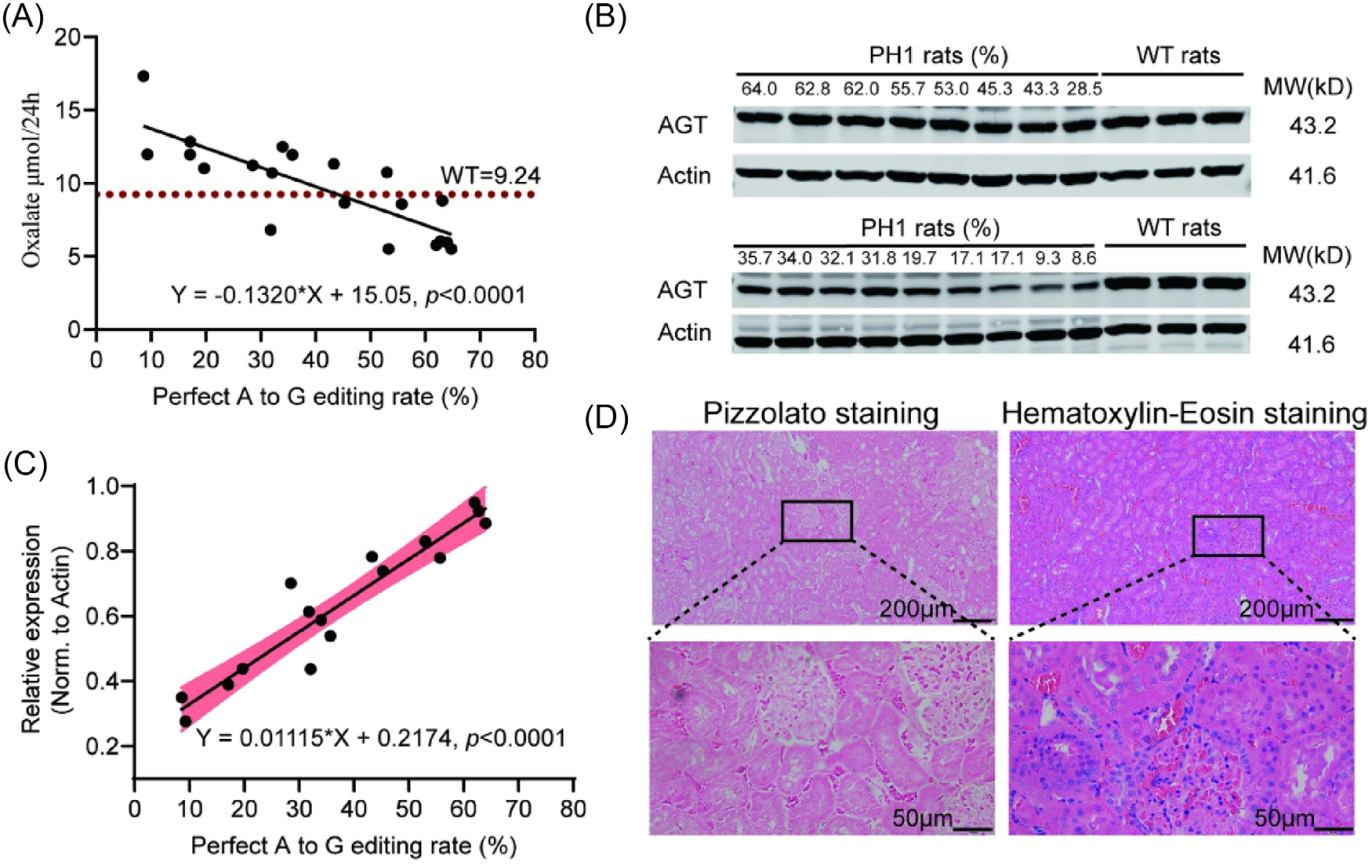
Therapeutic threshold of *Agxt* pathogenic variant correction in primary hyperoxaluria type 1 (PH1) rats. (A) Correlation of DNA correction rate with urinary oxalate level in 2‐month‐old PH1‐Corrected rats. (B) Western blot analysis of alanine‐glyoxylate aminotransferase (AGT) protein expression in liver tissues of PH1 rats with varying *Agxt* pathogenic variant correction efficiencies. (C) Correlation of DNA correction rate with relative AGT protein expression in 2‐month‐old PH1‐Corrected rats. The AGT band intensity was normalised to Actin and expressed relative to that of wild‐type (WT)‐Control rats. (D) Representative Pizzolato and haematoxylin‒eosin (H&E) staining of renal sections from a PH1 rat with 45.27% correction efficiency after the 10‐day ethylene glycol (EG) challenge.

Next, we sought to determine the minimal *Agxt* pathogenic variant correction proportion required to achieve phenotypic rescue. To expand the range of editing efficiencies, we supplemented the cohort described above with five additional PH1 rats receiving.35 mg/kg injections, resulting in a mean correction efficiency of 38.86% for *Agxt* pathogenic mutations (Figure S6A,B). The total cohort of 20 rats across four dosage groups (.25,.35,.5 and 1.0 mg/kg) exhibited *Agxt* correction efficiencies ranging from 9.2% to 68.1% (Figure 8A). Given the stabilisation of 24‐h urinary oxalate levels in adult PH1 rats, we assessed the 24‐h urinary oxalate levels at the age of 2 months and established the correlation between correction efficiency and UOx levels, as formalised in Figure 8A. The data showed that the UOx levels decreased with the increase of the *Agxt* genome correction rate, indicating a linear relationship (*R* ^2^ = .6495, *p* < .0001) with the equation *Y* = ‒.1320*X* + 15.05, where *Y* represented urinary oxalate levels and *X* represented editing efficiency (Figure 8A). Using age‐matched male WT rat UOx levels as normalisation thresholds, computational analysis revealed that ∼44% *Agxt* correction efficiency was required for oxalate normalisation.

Since the correction of *Agxt* pathogenic mutations ultimately restores AGT protein expression, we further investigated the minimum AGT expression threshold required for urinary oxalate normalisation. Using WT hepatic AGT levels as reference, we performed Western blot analysis to quantify relative AGT expression in PH1 rats with varied correction efficiencies (Figure 8B). Linear regression of AGT expression versus *Agxt* correction efficiency demonstrated efficiency‐dependent protein restoration (Figure 8C). Importantly, the established 44% correction threshold corresponded to 71% of WT AGT expression levels (Figure 8C). Histopathological validation through H&E and Pizzolato staining of renal tissue from a PH1 rat with 45.27% correction efficiency revealed no evidence of nephropathic changes or CaOx deposition following EG challenge (Figure 8D). These results suggested that approximately 44% *Agxt* correction efficiency constitutes the therapeutic threshold for the cure of PH1.

## 3 DISCUSSION

**FIGURE 9:**
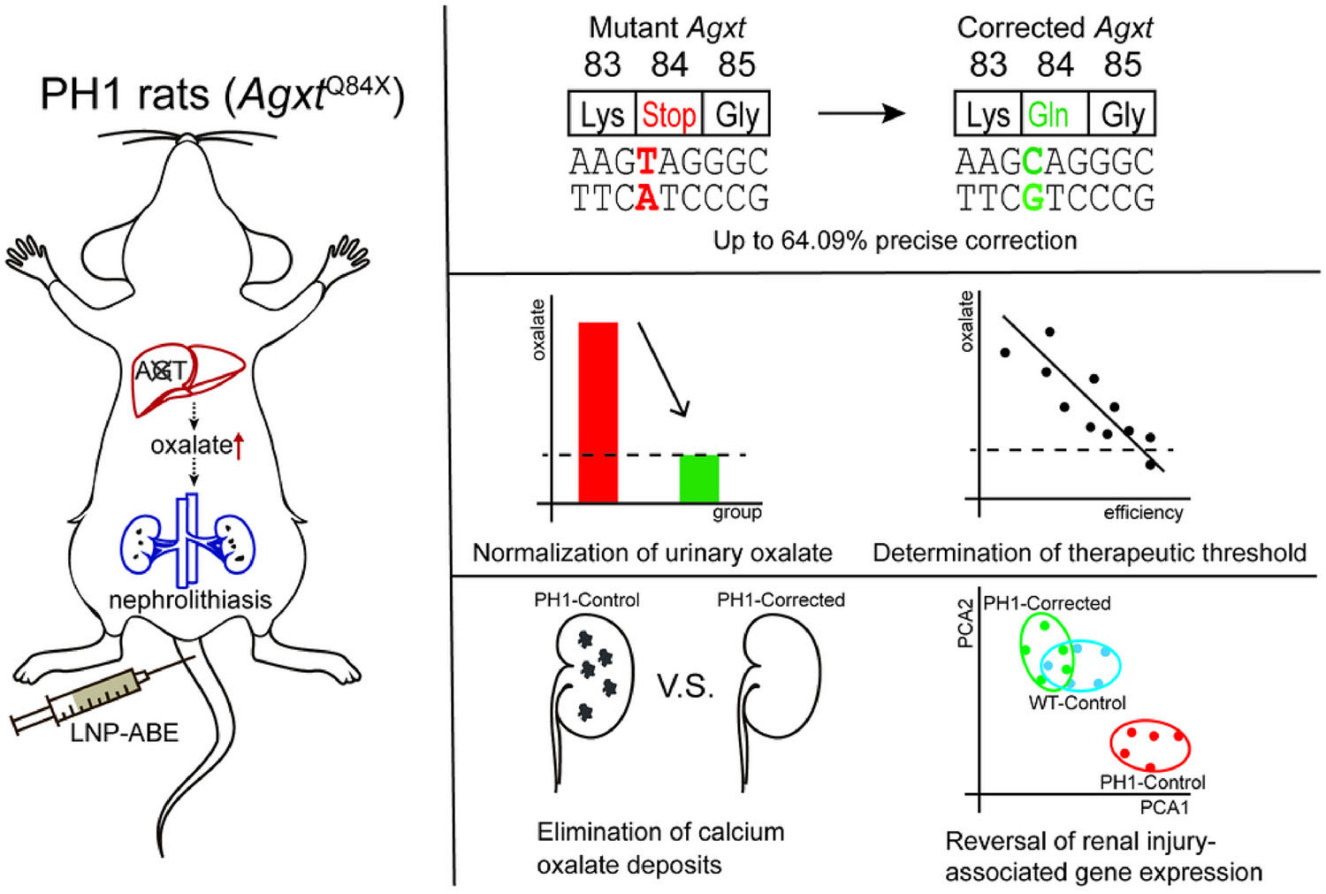
Graphical abstract of this work.

*AGXT* gene mutations are the primary etiology of PH1. Correcting *AGXT* pathogenic mutations in vivo through gene‐editing technologies to restore AGT protein expression represents an ideal therapeutic strategy. In this study, through systematic screening of base editors and dose optimisation in LNP‐delivered base editing systems, we achieved high‐efficiency correction of *Agxt* mutations in PH1 rats, restoring hepatic AGT protein to normal levels. This strategy successfully reduced urinary oxalate to physiological levels, eliminated renal CaOx deposition, rescued pathological alterations, reversed expression patterns of kidney injury‐related genes and modulated hepatic gene expression patterns (Figure 9). We also validated the therapeutic efficacy in adult PH1 rats. Furthermore, we systematically investigated the correlation between *Agxt* correction efficiency and urinary oxalate levels, establishing the minimal thresholds of *Agxt* correction efficiency (∼44%) and AGT protein expression (71% of WT levels) required for phenotypic normalisation, providing critical references for clinical translation.

Restoring the expression of AGT protein is the key focus in PH1 therapeutic research. Salido et al. utilised adenoviral vectors to deliver the human *AGXT* gene for PH1 mouse treatment, achieving significant urinary oxalate reduction at 7 and 14 days post‐treatment.^15^ Similarly, Yang et al. demonstrated that lipopolyplex‐delivered sequence‐optimised human AGT mRNA restored peroxisomal AGT enzyme function in PH1 rats, with a single 2 mg/kg dose inducing 70% reduction in urinary oxalate levels.^16^ However, the efficacy of these therapeutic interventions gradually diminished over time. To achieve durable PH1 treatment, researchers utilised gene‐editing strategies to restore endogenous *Agxt* expression. Nieto‐Romero et al. corrected *Agxt* mutations in patient‐derived cells through CRISPR/Cas9‐mediated homology‐directed repair, restoring glyoxylate metabolism.^18^ However, this study lacked in vivo therapeutic validation. Additionally, our previous studies demonstrated that AAV‐delivered base editors could rectify *Agxt* mutations in PH1 rats with therapeutic effects, but the limited correction efficiency in vivo failed to achieve a phenotypic cure of PH1.^24^ To enhance in vivo editing efficiency, this study employed LNP characterised by greater packaging capacity, reduced immunogenicity and potential for repeat administration.^27^, ^40^, ^41^ Through systematic screening of base editor variants and dosage optimisation, we achieved high‐efficiency *Agxt* mutation correction in PH1 rats, resulting in the phenotypic rescue of the disease.

An interesting finding in this study is the modulation of hepatic transcriptional programs by LNP‐ABE‐mediated *Agxt* mutation correction in PH1 rats. PH1‐corrected animals exhibited upregulated hepatic metabolic pathways, suggesting restoration of hepatic functional competency. This coordinated metabolic reprogramming was paralleled by significant downregulation of proinflammatory and immune markers, although the absence of overt histopathological alterations at this early intervention time point. Importantly, our findings align with prior reports that *AGXT* overexpression ameliorates diet‐induced MASH by enhancing hepatic metabolic flexibility,^42^ indicating that *Agxt* correction decreases susceptibility to hepatic injury.

PH1 patients diagnosed in adulthood represent a distinct clinical population characterised by delayed therapeutic intervention following prolonged hyperoxaluria.^6^ However, previous studies did not assess therapeutic interventions for PH1 populations with prolonged hyperoxaluria duration. Unlike PH1 mouse models, our PH1 rat model exhibits early‐onset symptoms,^43^ better recapitulating adult patients with delayed diagnosis. In this study, we demonstrated that adult‐onset treatment effectively normalised urinary oxalate and prevented nephrocalcinosis, highlighting the robustness of our therapeutic approach.

In gene‐editing therapies, editing efficiency is a critical determinant of therapeutic outcomes. Determination of the minimal therapeutic threshold not only clarifies the relative therapeutic potential of target genes but also provides a scientific foundation for clinical dosing regimen optimisation. Previous research suggested that >60% and >50% editing efficiency of *Hao1* gene^44^ and *Ldha* gene^45^ were required for PH1 cure. Since the PH1 rat produces no endogenous AGT protein, it provides a clean background for precisely evaluating the efficacy of our gene‐editing strategy. Our results showed that correcting the *Agxt* gene necessitated lower editing efficiency, indicating superior therapeutic efficacy of *Agxt* correction. This observation aligns with the biochemical characteristics of AGT protein, including high substrate affinity and catalytic capacity,^46^ wherein partial functional restoration enables effective glyoxylate metabolism. Although the specific mutation studied is not the most common, our study serves as an important demonstration that LNP‐delivered base editing can achieve robust therapeutic efficacy in vivo. A key translational insight from our work is the determination of a minimum editing efficiency threshold required for phenotypic rescue, a parameter that will be broadly informative for future clinical development of editing therapies for monogenic metabolic disorders, even beyond PH1.

Despite its therapeutic potential, the clinical translation of *AGXT* mutation correction encounters several challenges. The heterogeneity in mutation types and loci requires case‐specific design of sgRNAs, selection of appropriate base editors, and comprehensive safety validation for individual variants. Furthermore, base editing was unable to correct *AGXT* indel mutations in a subset of PH1 patients. These limitations constrain the universal applicability of *AGXT* base editing therapies. Compared with base editors, prime editors^47^ demonstrate the capacity to modify a broad spectrum of genetic mutations, including indels, emerging as a promising therapeutic modality for PH1 management, although their editing efficiency still lags behind that of base editors. What's more, the emergence of recombinase‐, integrase‐ and retrotransposon‐associated gene‐editing platforms^48^, ^49^, ^50^ has enabled site‐specific integration of the targeted gene, potentially offering a universal therapeutic strategy for PH1. In addition, advances in delivery strategies, such as virus‐like particles and biodegradable LNPs, hold promise for further improving the safety and efficacy of this therapeutic approach.^51^, ^52^ Furthermore, comprehensive safety profiling of this therapeutic strategy is essential. Monitoring both hepatotoxicity and immune responses following LNP‐mRNA administration at early time points is critical for successful clinical translation.

In conclusion, this study establishes LNP‐delivered base editing the *Agxt* mutations as a curative strategy for PH1 in rat models, validates its efficacy in adult‐onset treatment, and defines critical therapeutic thresholds. However, the translational relevance of these findings requires verification in PH1 patients. Future studies should address mutation diversity challenges and optimise platform versatility for broader clinical applications.

## 4 METHODS AND MATERIALS

### 4.1 Plasmids construction

All ABE variant expression plasmids utilised in this study were constructed using standard molecular cloning techniques. Plasmids encoding ABEmax and ABE8e were acquired from Addgene (plasmid numbers 112095 and 138489). The VQR‐spCas9, spG‐spCas9, spNG‐spCas9 and spRY‐spCas9 variants^53^, ^54^, ^55^ were incorporated via overlap extension polymerase chain reaction (PCR). The coding sequence of ABE was cloned into an mRNA production plasmid downstream of a T7 promoter for mRNA synthesis.

### 4.2 RNA production

In vitro transcription and purification with Co‐capping T7 in vitro transcription reagent (Hzymes HBP001509) was used to generate the Luciferase and ABE mRNA transcript. Linearised plasmid DNA with a 120 nt poly(A) tail served as the template and the UTP was substituted with N1‐methylpseudouridine. mRNA integrity was verified by capillary electrophoresis, and concentration was quantified via NanoDrop spectrophotometry. The sgRNA used in this study, which was chemically synthesised with end‐modifications (2′‐O‐methylation and phosphorothioate linkage), was purchased from Genscript: mG*mC*mC*CUACUUGAUCUCAUCCAGUUUUAGAGCUAGAAAUAGCAAGUUAAAAUAAGGCUAGUCCGUUAUCAACUUGAAAAAGUGGCACCGAGUCGGUGCU*mU*mU*mU.

### 4.3 LNP encapsulation

Purified mRNA and the synthesised sgRNA were encapsulated into LNPs as described previously.^56^ Briefly, an ethanol solution of ALC‐0315 (Sinopharm TMLT9277MG1), DSPC (Sinopharm XW0281694401), cholesterol (Sinopharm XW01214046704) and PEG lipid (Sinopharm 30151428) (at molar ratios of 46:10:42.5:1.5) was rapidly mixed with a sodium acetate solution (pH 4) containing mRNA and sgRNA (at a weight ratio 1:1) using a Precision Nanosystems NanoAssemblr Benchtop Instrument. Then, the LNP formulation was dialysed against PBS (pH 7.4) and sterile‐filtered using.2‐µm filters. LNP had an average particle size of 90 nm, with a polydispersity index of <.15 as determined by dynamic light scattering.

### 4.4 Cell culture and transfection

HEK293T cells (ATCC) were maintained in Dulbecco's Modified Eagle's Medium (DMEM) (Gibco 11965092) supplemented with 10% foetal bovine serum (Yeasen 40131ES76) and 1% penicillin‒streptomycin at 37°C under 5% CO_2_. HEK293T cells were seeded on 24‐well plates at 3.0 × 10^5^ cells per well. Cells were transfected with plasmids using polyethyleneimine (Polysciences) at approximately 80% confluency. Cells were transfected with RNA using Lipofectamine 3000 (Invitrogen L3000001) at approximately 50% confluency. Cells were cultured for 72 h after transfection, and then genomic DNA was isolated for further analysis.

### 4.5 Animal studies

All animal procedures were approved by the Association for Assessment and Accreditation of Laboratory Animal Care in Shanghai and the Laboratory Animal Center Fudan University (approval 2024‐EKYY‐015). The *Agxt^Q84X^ PH1* Sprague‒Dawley (c.250 C>T, p.Q84X) rats were obtained as previously described. The *Agxt^Q84X^ PH1* rat carries a nonsense mutation that results in a complete lack of AGT protein expression and develops robust hyperoxaluria early in life, closely mirroring the aggressive disease phenotype observed in a significant subset of PH1 patients. Only male rats were used in this study. All PH1 and WT rats used in this study were housed under standard conditions (12‐h light/dark cycle) with sufficient food and water.

For in vivo biodistribution analysis of LNPs, 3‐week‐age WT rats were administered with LNP‐Luciferase formulations via tail vein at a dose of.1 mg/kg (*n* = 3). The degree and biodistribution of luciferase were detectable by bioluminescence imaging at 6, 24 and 48 h after LNP‐Luciferase injection using an IVIS system (PerkinElmer). For *Agxt* mutation correction, 3‐week‐age PH1 male rats were randomly divided into several groups and administered with LNP‐ABE via tail at a dose of.25‒1.0 mg/kg, and age‐matched PH1 and WT rats received PBS were served as controls (*n* = 5 per group). Liver biopsy was performed 1 week after the injection. At the ages of 1, 2, 3, 4, 5 and 6 months, all rats were individually housed in metabolic cages for 24‐h urine collection. At the age of 6 months, all rats were given.7% (v/v) EG water for 10 days. During the challenge, all rats were individualised housed in metabolic cages for the collection of 24 h urine and monitoring of water intake. Twenty‐four‐hour urine was collected during the last day of the EG challenge. After the EG challenge, rats were euthanised by carbon dioxide inhalation, and blood samples and several tissues, including heart, liver, spleen, kidneys, lungs, skeletal muscle and testes, were collected.

### 4.6 RNA isolation and RT‐qPCR

RNA isolation was performed using the MolPure TRIeasy Plus Total RNA Kit (Yeasen 19211ES60), according to the protocol of the manufacturer. cDNA was reverse transcribed using the Hifair II 1st Strand cDNA Synthesis Kit (Yeasen 11119ES60). RT‐PCR was performed using the Hieff qPCR SYBR Green Master Mix (Yeasen 11202ES03) with gene‐specific primers (Table S4) and analysed by QuantStudio Real‐Time PCR System (Thermo Fisher Scientific). Relative fold changes (FCs) were determined using the ΔΔCt method.

### 4.7 Western blot analysis

Total proteins of rat liver or cells were extracted with RIPA buffer and quantified using the BCA Protein Quantification Kit (Yeasen 20201ES76). An amount of 30 µg of total protein with loading buffer was loaded in 10% SDS‒PAGE gels and transferred to a nitrocellulose membrane. Then, membranes were blocked with 5% bovine serum albumin (BSA; Servicebio GC305010) for 1 h at room temperature on a shaker. Next, membranes were probed with primary antibodies (Novus Biologicals, NBP1‐89200, 1:5000) or a mouse anti‐actin antibody (Sigma, 1:5000) overnight at 4°C, followed by corresponding secondary antibodies for 1 h at room temperature. Members were scanned by Odyssey Fc (LI‐COR) imaging system for image generation.

### 4.8 Histological analysis

For H&E staining, paraffin sections were stained with H&E solution. For CaOx crystal detection, Pizzolato staining was performed by incubating sections with a mixing solution of 5% silver nitrate and 30% hydrogen peroxide under ultraviolet light (30 min). For immunohistochemistry, sections were blocked with 5% BSA for 1 h at room time and incubated overnight with primary antibodies (Novus Biologicals, NBP1‐89200). Slides were stained using a Pierce DAB (Thermo 34002). Slides were examined for histopathological changes and CaOx deposition levels using polarised light microscopy.

### 4.9 Urinary oxalate detection

Urine samples were collected over 24 h using metabolic cages, acidified with HCl, and filtered using.45‐µm filters. Oxalate levels were measured using ion exchange chromatography (Dionex ICS‐5000, Thermo Fisher Scientific). Specifically, the quantification of urinary oxalate in rats was performed by a technician who was blinded to the experimental group assignments. Twenty‐four‐hour urinary oxalate level was calculated based on the oxalate concentration and the volume of the urine sample.

### 4.10 Serum analysis

Blood samples were collected via retro‐orbital venous plexus bleeding at various time points, incubated overnight at 4°C for 2 h, and centrifuged at 12 000 rpm at 4°C for 15 min to isolate serum. ALT, AST, BUN and CREA levels were measured by Servicebio.

### 4.11 Next‐generation sequencing

Total genomic DNA was extracted from tissues or cells using TIANamp Genomic DNA kit (Tiangen DP304) according to the standard protocols. The first‐round PCR reactions were performed using 2×Hieff PCR Master Mix (Yeasen 10102ES03) and the primer sets (Table S5) to amplify the targeted region of interest. A secondary barcoding PCR was conducted using the above PCR products and specific primers with different barcode sequences. After purification and normalisation, final pooled libraries were sequenced on the Illumina Hiseq platform. The data were analysed with CRISPR RGEN Tools.^57^ On‐target base editing efficiencies on A6, A11, A16 and A20 within the sgRNA were quantified as percentage of (number of reads containing the desired edit)/(number of total aligned reads). Perfect editing efficiency was determined by the ratio of (the number of reads containing only A6‐to‐G modification)/(total aligned reads). Off‐target base editing rates were calculated as the percentage of (the number of reads containing A‐to‐G modifications throughout the candidate off‐target sequences)/(total aligned reads).

### 4.12 Bulk RNA sequencing

According to the manufacturer's protocol, rat liver or kidney RNA was extracted using the MolPure TRIeasy Plus Total RNA Kit (Yeasen 19211ES60) and sequenced on the Illumina Novaseq 6000 (LC Bio Technology Co. Ltd.). The DEGs were identified using Cufflinks (|log2 FC| > 1, *p* < .05) and annotated via KEGG/GO enrichment analysis.

### 4.13 Whole‐genome sequencing

WGS was performed to assess potential off‐target editing events. Sequencing libraries were prepared from genomic DNA isolated from the liver tissues of 6‐month‐old PH1‐Corrected rats and PH1‐Control rats (*n* = 3). All libraries were sequenced on the Illumina platform to achieve an average depth of 30× coverage across the genome.

To quantify off‐target effects, the frequency of SNVs was calculated as the ratio of the number of variant‐supporting reads to the total number of sequenced bases for each sample. The SNV frequency in the PH1‐Corrected rats was then statistically compared to that in the PH1‐Control rats to determine if there was a significant increase in off‐target editing attributable to the gene‐editing procedure.

### 4.14 Statistics

All data are represented as mean ± SEM. Differences were assessed using two‐tailed Student's *t*‐test or by one‐way analysis of variance (ANOVA). The quantification of urinary oxalate in rats was performed by a technician who was blinded to the experimental group assignments. The subsequent data analysis was conducted by the experimenters without blinding. The normality of continuous variables was assessed using the Shapiro‒Wilk test and visually verified by Q‒Q plots. All analysed variables met the normality assumption. Prism software (GraphPad) was used for comparison between groups. A *p*‐value of less than.05 was considered significant.

## AUTHOR CONTRIBUTIONS

Hongquan Geng and Dali Li supervised the research and obtained funding. Dexin Zhang and Rui Zheng performed most experiments with the help of Xi Chen, Lei Yang, Yanan Huo, Zhoutong Chen, Jiaxin Huang, Yining Zhao and Shuming Yin. Dan Zhang performed next‐generation sequencing analysis. Dexin Zhang wrote the manuscript. All the authors contributed to editing the manuscript.

## CONFLICT OF INTEREST STATEMENT

The authors declare they have no conflicts of interest.

## ETHICS STATEMENT

All animal procedures were approved by the Association for Assessment and Accreditation of Laboratory Animal Care in Shanghai and the Laboratory Animal Center Fudan University (approval2024‐EKYY‐015).

## Supporting information



Supporting Information

## Data Availability

The deep‐sequencing data have been deposited in the NCBI Sequence Read Archive database under accession code PRJNA1256093.

## References

[1] Milliner DS , Harris PC , Sas DJ , Cogal AG . GeneReviews is a registered trademark of the University of Washington. In: Adam MP , Feldman J , Mirzaa GM , Pagon RA , Wallace SE , Amemiya A , eds. GeneReviews®. Seattle Copyright © 1993‐2025, University of Washington; 1993.

[2] Danpure CJ , Jennings PR , Watts RW . Enzymological diagnosis of primary hyperoxaluria type 1 by measurement of hepatic alanine: glyoxylate aminotransferase activity. Lancet. 1987;1(8528):289‐291. 2880111 10.1016/s0140-6736(87)92023-x

[3] Groothoff JW , Metry E , Deesker L , et al. Clinical practice recommendations for primary hyperoxaluria: an expert consensus statement from ERKNet and OxalEurope. Nat Rev Nephrol. 2023;19(3):194‐211. 36604599 10.1038/s41581-022-00661-1

[4] Rumsby G , Cochat P . Primary hyperoxaluria. N Engl J Med. 2013;369(22):2163. 10.1056/NEJMc1311606 24283239

[5] Mandrile G , van Woerden CS , Berchialla P , et al. Data from a large European study indicate that the outcome of primary hyperoxaluria type 1 correlates with the AGXT mutation type. Kidney Int. 2014;86(6):1197‐1204. 24988064 10.1038/ki.2014.222

[6] Metry EL , Garrelfs SF , Deesker LJ , et al. Determinants of kidney failure in primary hyperoxaluria type 1: findings of the European hyperoxaluria consortium. Kidney Int Rep. 2023;8(10):2029‐2042. 37849991 10.1016/j.ekir.2023.07.025 PMC10577369

[7] Pszczolinski R , Acquaviva C , Berrahal I , et al. Primary hyperoxaluria in adults and children: a nationwide cohort highlights a persistent diagnostic delay. Clin Kidney J. 2024;17(5):sfae099. 38737343 10.1093/ckj/sfae099 PMC11087826

[8] Michael M , Harvey E , Milliner DS , et al. Diagnosis and management of primary hyperoxalurias: best practices. Pediatr Nephrol. 2024;39(11):3143‐3155. 38753085 10.1007/s00467-024-06328-2

[9] Cellini B , Montioli R , Oppici E , Astegno A , Voltattorni CB . The chaperone role of the pyridoxal 5′‐phosphate and its implications for rare diseases involving B6‐dependent enzymes. Clin Biochem. 2014;47(3):158‐165. 10.1016/j.clinbiochem.2013.11.021 24355692

[10] van Woerden CS , Groothoff JW , Wijburg FA , Annink C , Wanders RJ , Waterham HR . Clinical implications of mutation analysis in primary hyperoxaluria type 1. Kidney Int. 2004;66(2):746‐752. 15253729 10.1111/j.1523-1755.2004.00796.x

[11] Hoyer‐Kuhn H , Kohbrok S , Volland R , et al. Vitamin B6 in primary hyperoxaluria I: first prospective trial after 40 years of practice. Clin J Am Soc Nephrol. 2014;9(3):468‐477. 24385516 10.2215/CJN.06820613 PMC3944765

[12] Shah VN , Pyle L . Lumasiran, an RNAi therapeutic for primary hyperoxaluria type 1. N Engl J Med. 2021;385(20):e69. 10.1056/NEJMc2107661 34758264

[13] Hoppe B , Koch A , Cochat P , et al. Safety, pharmacodynamics, and exposure‐response modeling results from a first‐in‐human phase 1 study of nedosiran (PHYOX1) in primary hyperoxaluria. Kidney Int. 2022;101(3):626‐634. 34481803 10.1016/j.kint.2021.08.015

[14] Metry EL , van Dijk LMM , Peters‐Sengers H , et al. Transplantation outcomes in patients with primary hyperoxaluria: a systematic review. Pediatr Nephrol. 2021;36(8):2217‐2226. 33830344 10.1007/s00467-021-05043-6 PMC8260423

[15] Salido EC , Li XM , Lu Y , et al. Alanine‐glyoxylate aminotransferase‐deficient mice, a model for primary hyperoxaluria that responds to adenoviral gene transfer. Proc Natl Acad Sci U S A. 2006;103(48):18249‐18254. 17110443 10.1073/pnas.0607218103 PMC1838738

[16] Yang T , Ge J , Huang L , et al. Preclinical evaluation of AGT mRNA replacement therapy for primary hyperoxaluria type I disease. Sci Adv. 2025;11(15):eadt9694. 40203111 10.1126/sciadv.adt9694 PMC11980851

[17] Wang L , Zhou B , Li D . Genome editing technology and medical applications. Sci China Life Sci. 2024;67(12):2537‐2539. 39560684 10.1007/s11427-024-2773-3

[18] Nieto‐Romero V , García‐Torralba A , Molinos‐Vicente A , et al. Restored glyoxylate metabolism after AGXT gene correction and direct reprogramming of primary hyperoxaluria type 1 fibroblasts. iScience. 2024;27(4):109530. 38577102 10.1016/j.isci.2024.109530 PMC10993186

[19] Komor AC , Badran AH , Liu DR . CRISPR‐based technologies for the manipulation of eukaryotic genomes. Cell. 2017;168(1‐2):20‐36. 27866654 10.1016/j.cell.2016.10.044 PMC5235943

[20] Yin S , Gao L , Sun X , et al. Amelioration of metabolic and behavioral defects through base editing in the Pah(R408W) phenylketonuria mouse model. Mol Ther. 2025;33(1):119‐132. 39600089 10.1016/j.ymthe.2024.11.032 PMC11764323

[21] Li WK , Zhang SQ , Peng WL , et al. Whole‐brain in vivo base editing reverses behavioral changes in Mef2c‐mutant mice. Nat Neurosci. 2024;27(1):116‐128. 38012399 10.1038/s41593-023-01499-x

[22] Cui C , Wang S , Wang D , et al. A base editor for the long‐term restoration of auditory function in mice with recessive profound deafness. Nat Biomed Eng. 2025;9(1):40‐56. 39134683 10.1038/s41551-024-01235-1

[23] Kabra M , Shahi PK , Wang Y , et al. Nonviral base editing of KCNJ13 mutation preserves vision in a model of inherited retinal channelopathy. J Clin Invest. 2023;133(19):e171356. 10.1172/JCI171356 PMC10541187 37561581

[24] Chen Z , Zhang D , Zheng R , et al. In vivo base editing rescues primary hyperoxaluria type 1 in rats. Kidney Int. 2024;105(3):496‐507. 38142039 10.1016/j.kint.2023.11.029

[25] Yang L , Wang L , Huo Y , et al. Amelioration of an inherited metabolic liver disease through creation of a de novo start codon by cytidine base editing. Mol Ther. 2020;28(7):1673‐1683. 32413280 10.1016/j.ymthe.2020.05.001 PMC7335753

[26] Keeler AM , Zhan W , Ram S , Fitzgerald KA , Gao G . The curious case of AAV immunology. Mol Ther. 2025;33:1946‐1965. 10.1016/j.ymthe.2025.03.037 PMC12126790 40156190

[27] van Haasteren J , Li J , Scheideler OJ , Murthy N , Schaffer DV . The delivery challenge: fulfilling the promise of therapeutic genome editing. Nat Biotechnol. 2020;38(7):845‐855. 32601435 10.1038/s41587-020-0565-5

[28] Raguram A , Banskota S , Liu DR . Therapeutic in vivo delivery of gene editing agents. Cell. 2022;185(15):2806‐2827. 35798006 10.1016/j.cell.2022.03.045 PMC9454337

[29] Li Y , Zheng R , Xu G , et al. Generation and characterization of a novel rat model of primary hyperoxaluria type 1 with a nonsense mutation in alanine‐glyoxylate aminotransferase gene. Am J Physiol Renal Physiol. 2021;320(3):F475‐F484. 33491567 10.1152/ajprenal.00514.2020

[30] Choi Y , Chan AP . PROVEAN web server: a tool to predict the functional effect of amino acid substitutions and indels. Bioinformatics. 2015;31(16):2745‐2747. 25851949 10.1093/bioinformatics/btv195 PMC4528627

[31] Musunuru K , Chadwick AC , Mizoguchi T , et al. In vivo CRISPR base editing of PCSK9 durably lowers cholesterol in primates. Nature. 2021;593(7859):429‐434. 34012082 10.1038/s41586-021-03534-y

[32] Gianmoena K , Gasparoni N , Jashari A , et al. Epigenomic and transcriptional profiling identifies impaired glyoxylate detoxification in NAFLD as a risk factor for hyperoxaluria. Cell Rep. 2021;36(8):109526. 34433051 10.1016/j.celrep.2021.109526

[33] Rom O , Liu Y , Liu Z , et al. Glycine‐based treatment ameliorates NAFLD by modulating fatty acid oxidation, glutathione synthesis, and the gut microbiome. Sci Transl Med. 2020;12(572):eaaz2841. 10.1126/scitranslmed.aaz2841 PMC7982985 33268508

[34] Recker P , Beck BB , Sikora P , et al. Chronic liver disease and hepatic calcium‐oxalate deposition in patients with primary hyperoxaluria type I. Sci Rep. 2022;12(1):16725. 36202824 10.1038/s41598-022-19584-9 PMC9537520

[35] Brooks DL , Carrasco MJ , Qu P , et al. Rapid and definitive treatment of phenylketonuria in variant‐humanized mice with corrective editing. Nat Commun. 2023;14(1):3451. 37301931 10.1038/s41467-023-39246-2 PMC10257655

[36] Rothgangl T , Dennis MK , Lin PJC , et al. In vivo adenine base editing of PCSK9 in macaques reduces LDL cholesterol levels. Nat Biotechnol. 2021;39(8):949‐957. 34012094 10.1038/s41587-021-00933-4 PMC8352781

[37] Bae S , Park J , Kim JS . Cas‐OFFinder: a fast and versatile algorithm that searches for potential off‐target sites of Cas9 RNA‐guided endonucleases. Bioinformatics. 2014;30(10):1473‐1475. 24463181 10.1093/bioinformatics/btu048 PMC4016707

[38] Karlsson M , Zhang C , Méar L , et al. A single‐cell type transcriptomics map of human tissues. Sci Adv. 2021;7(31):eabh2169. 34321199 10.1126/sciadv.abh2169 PMC8318366

[39] Tang K , Ye T , He Y , et al. Ferroptosis, necroptosis, and pyroptosis in calcium oxalate crystal‐induced kidney injury. Biochim Biophys Acta Mol Basis Dis. 2025;1871(5):167791. 40086520 10.1016/j.bbadis.2025.167791

[40] Xu SJ , Hu ZZ , Song FL , Xu Y , Han XX . Lipid nanoparticles: composition, formulation, and application. Mol Ther Methods Clin Develop. 2025;33(2):101463. 10.1016/j.omtm.2025.101463 PMC12415982 40927763

[41] Wu F , Li N , Xiao Y , et al. Lipid nanoparticles for delivery of CRISPR gene editing components. Small Methods. 2025:e2401632. 40434188 10.1002/smtd.202401632 PMC12825352

[42] Das S , Finney AC , Anand SK , et al. Inhibition of hepatic oxalate overproduction ameliorates metabolic dysfunction‐associated steatohepatitis. Nat Metab. 2024;6(10):1939‐1962. 39333384 10.1038/s42255-024-01134-4 PMC11495999

[43] Zheng R , Li Y , Wang L , et al. CRISPR/Cas9‐mediated metabolic pathway reprogramming in a novel humanized rat model ameliorates primary hyperoxaluria type 1. Kidney Int. 2020;98(4):947‐957. 32464217 10.1016/j.kint.2020.04.049

[44] Zhang D , Zheng R , Chen Z , et al. Lipid nanoparticle‐mediated base‐editing of the Hao1 gene achieves sustainable primary hyperoxaluria type 1 therapy in rats. Sci China Life Sci. 2024;67(12):2575‐2586. 39425833 10.1007/s11427-024-2697-3

[45] Martinez‐Turrillas R , Martin‐Mallo A , Rodriguez‐Diaz S , et al. In vivo CRISPR‐Cas9 inhibition of hepatic LDH as treatment of primary hyperoxaluria. Mol Ther Methods Clin Dev. 2022;25:137‐146. 35402636 10.1016/j.omtm.2022.03.006 PMC8971349

[46] Ichiyama A . Studies on a unique organelle localization of a liver enzyme, serine:pyruvate (or alanine:glyoxylate) aminotransferase. Proc Jpn Acad Ser B Phys Biol Sci. 2011;87(5):274‐286. 10.2183/pjab.87.274 PMC3165904 21558762

[47] Chen PJ , Liu DR . Prime editing for precise and highly versatile genome manipulation. Nat Rev Genet. 2023;24(3):161‐177. 36344749 10.1038/s41576-022-00541-1 PMC10989687

[48] Yarnall MTN , Ioannidi EI , Schmitt‐Ulms C , et al. Drag‐and‐drop genome insertion of large sequences without double‐strand DNA cleavage using CRISPR‐directed integrases. Nat Biotechnol. 2023;41(4):500‐512. 36424489 10.1038/s41587-022-01527-4 PMC10257351

[49] Pandey S , Gao XD , Krasnow NA , et al. Efficient site‐specific integration of large genes in mammalian cells via continuously evolved recombinases and prime editing. Nat Biomed Eng. 2025;9(1):22‐39. 38858586 10.1038/s41551-024-01227-1 PMC11754103

[50] Fell CW , Villiger L , Lim J , et al. Reprogramming site‐specific retrotransposon activity to new DNA sites. Nature. 2025;642:1080‐1089. 10.1038/s41586-025-08877-4 40205048

[51] Han X , Xu J , Xu Y , et al. In situ combinatorial synthesis of degradable branched lipidoids for systemic delivery of mRNA therapeutics and gene editors. Nat Commun. 2024;15(1):1762. 38409275 10.1038/s41467-024-45537-z PMC10897129

[52] Han X , Xu Y , Ricciardi A , et al. Plug‐and‐play assembly of biodegradable ionizable lipids for potent mRNA delivery and gene editing in vivo. bioRxiv. 2025. 10.1073/pnas.2528144123 PMC13229237 42190011

[53] Walton RT , Christie KA , Whittaker MN , Kleinstiver BP . Unconstrained genome targeting with near‐PAMless engineered CRISPR‐Cas9 variants. Science. 2020;368(6488):290‐296. 32217751 10.1126/science.aba8853 PMC7297043

[54] Kleinstiver BP , Prew MS , Tsai SQ , et al. Engineered CRISPR‐Cas9 nucleases with altered PAM specificities. Nature. 2015;523(7561):481‐485. 26098369 10.1038/nature14592 PMC4540238

[55] Nishimasu H , Shi X , Ishiguro S , et al. Engineered CRISPR‐Cas9 nuclease with expanded targeting space. Science. 2018;361(6408):1259‐1262. 30166441 10.1126/science.aas9129 PMC6368452

[56] Finn JD , Smith AR , Patel MC , et al. A single administration of CRISPR/Cas9 lipid nanoparticles achieves robust and persistent in vivo genome editing. Cell Rep. 2018;22(9):2227‐2235. 29490262 10.1016/j.celrep.2018.02.014

[57] Hwang GH , Park J , Lim K , et al. Web‐based design and analysis tools for CRISPR base editing. BMC Bioinform. 2018;19(1):542. 10.1186/s12859-018-2585-4 PMC6307267 30587106

