# Supplementary material for: LNP‐mediated in vivo base editing corrects Agxt to cure primary hyperoxaluria type 1
Source: Clin Transl Med. 2025 Nov 23;15(11):e70533. doi: 10.1002/ctm2.70533 (PMC12640612; doi:10.1002/ctm2.70533)
Supplement: Supplementary file 1 — Supporting Information [file CTM2-15-e70533-s001.docx]

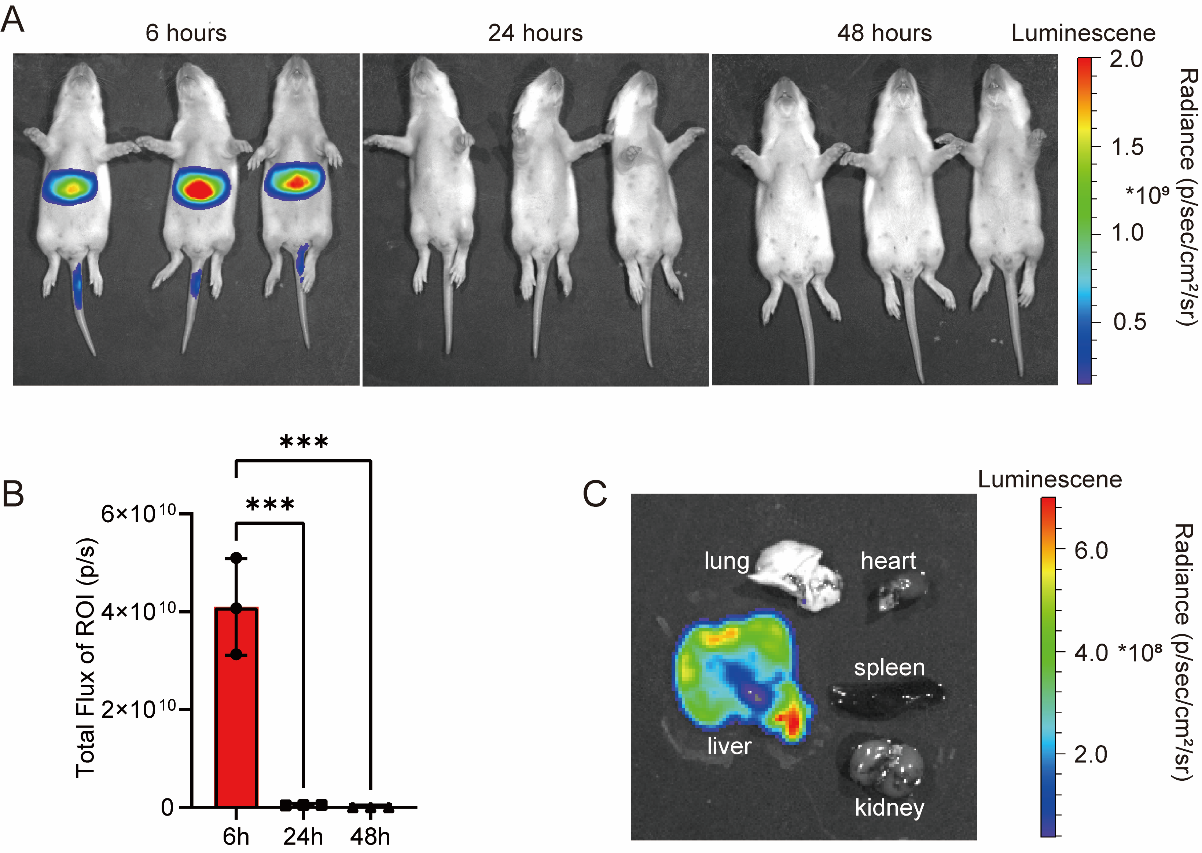


**Supplemental Figure 1.** The biodistribution pattern of the LNP-mRNA system used in this study.

(A) Distribution and intensity of luciferase expression at 6, 24, and 48 h after tail vein injection of 3-week-age rats with LNP-Luciferase was detected and quantified by bioluminescence imaging. (B) Quantitative analysis of bioluminescent signal intensities across time points. (C) *Ex vivo* bioluminescence imaging of heart, liver, spleen, lung, and kidney tissues harvested 6 hours post-LNP-Luciferase administration. Data are mean (SD). *p* values were calculated using One-way ANOVA. Ns, *p*>0.05; *, *p* < 0.05; **, *p*< 0.005; ***, *p* < 0.0005; ****, *p* < 0.0001.


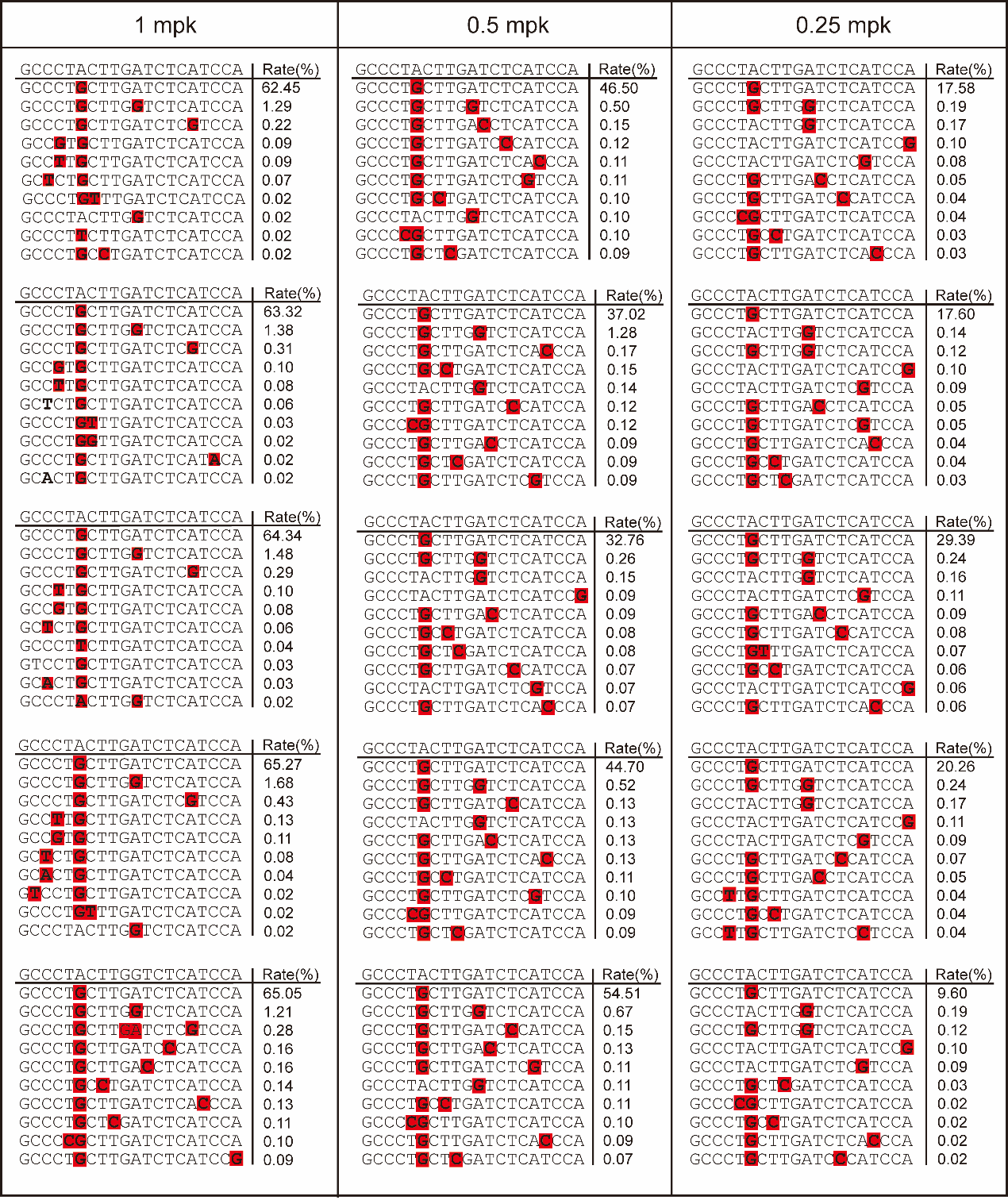


**Supplemental Figure 2.** Specific alleles with base substitution in each LNP-ABE treated PH1 rat. Only the top 10 alleles are listed.


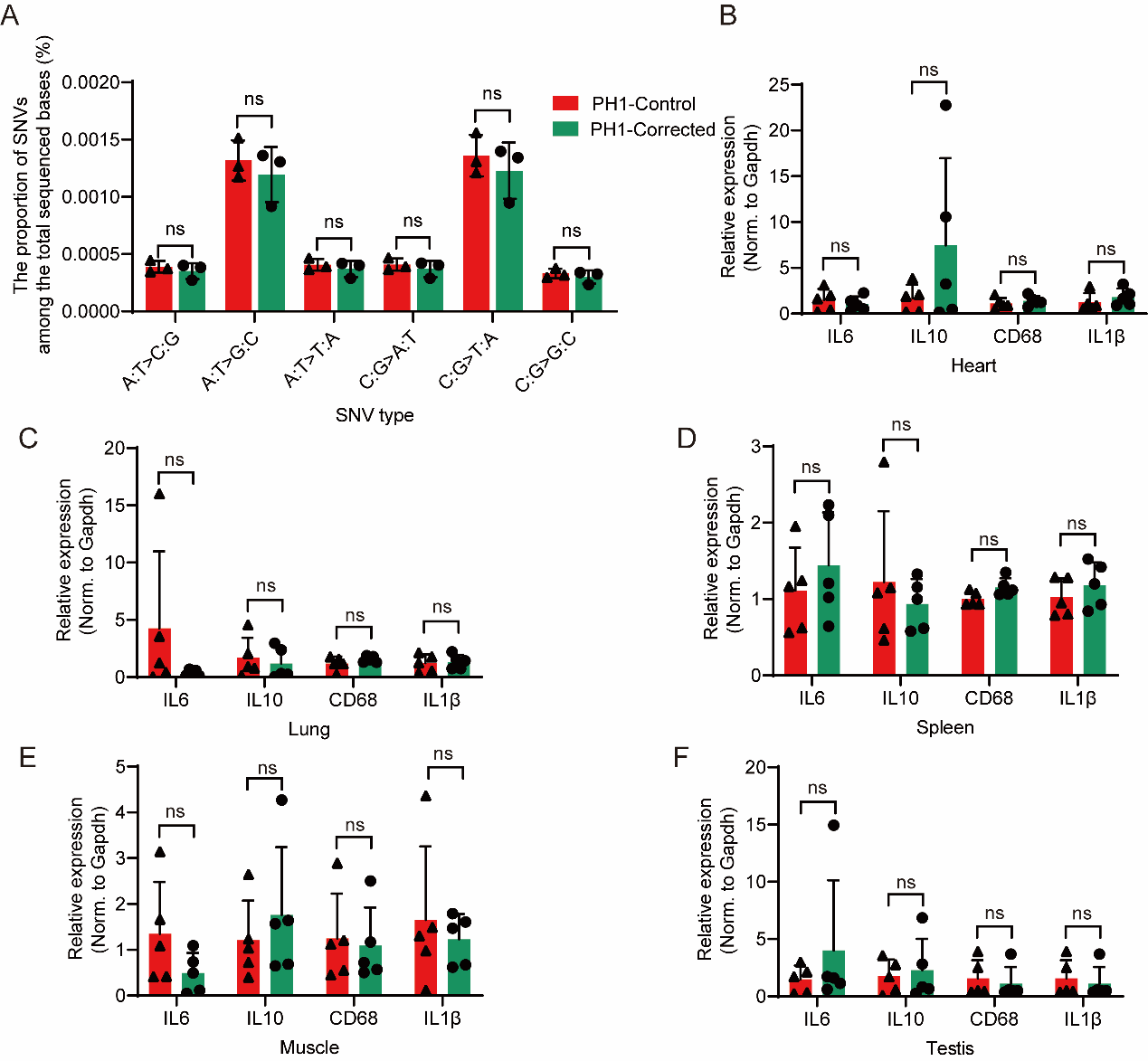


**Supplemental Figure 3. (A)** The proportion of single-base substitutions among the total sequenced bases of PH1-Control and PH1-Corrected rats (n = 3). RT-qPCR analysis of relative IL6, IL10, CD68, IL1β expression levels in the heart **(B)**, lung **(C)**, spleen **(D)**, muscle **(E)**, testis **(F)** tissues PH1-Control and PH1-Corrected rats (n=5). Data are mean (SD). *p* values were calculated using two-tailed unpaired t-tests. ns, *p*>0.05; *, p < 0.05; **, p< 0.005; ***, p < 0.0005; ****, p < 0.0001.


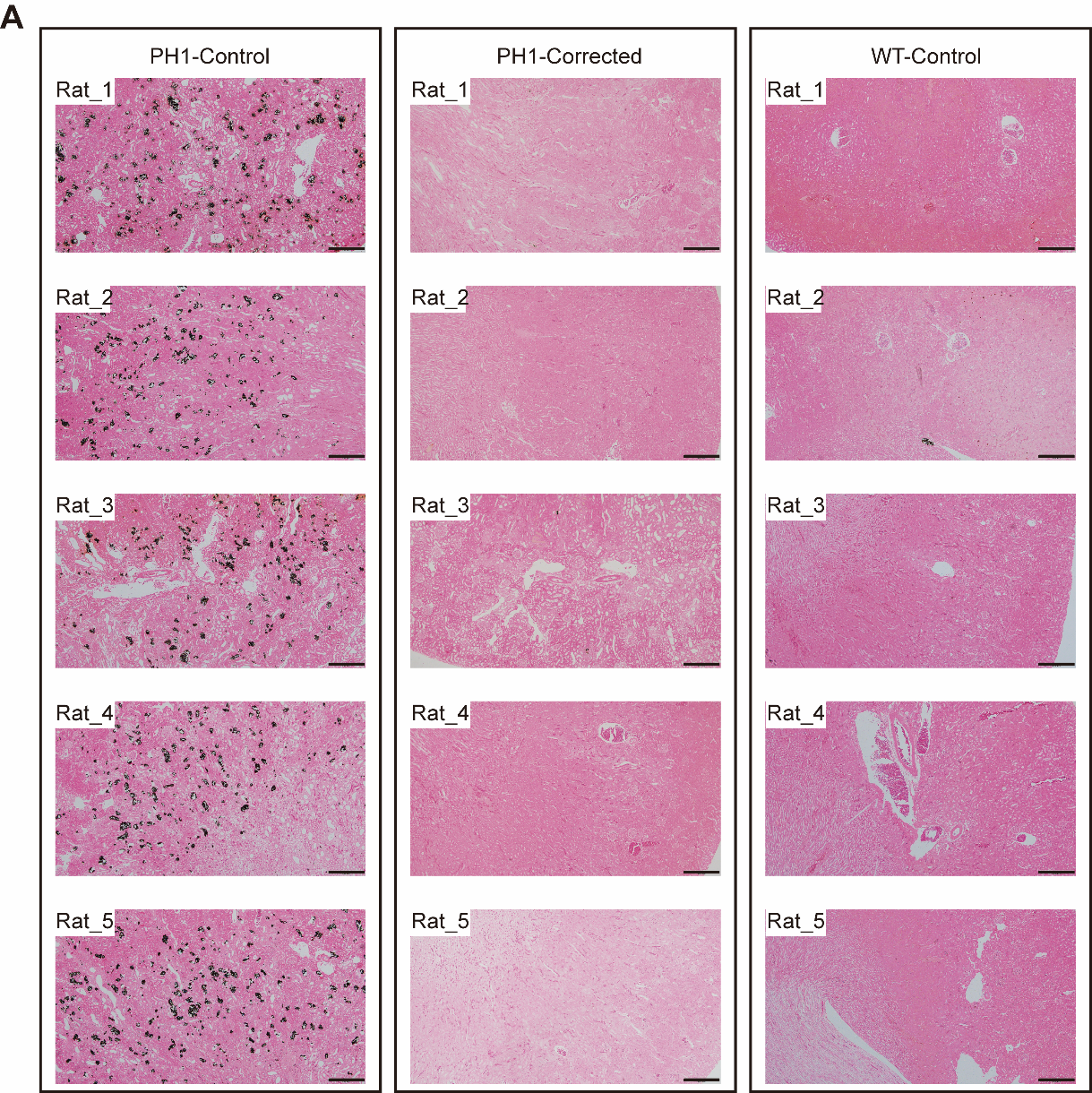


**Supplemental Figure 4.** Representative Pizzolato staining of renal sections from PH1-Control, PH1-Corrected, and WT-Control rats after the 10-day EG challenge.


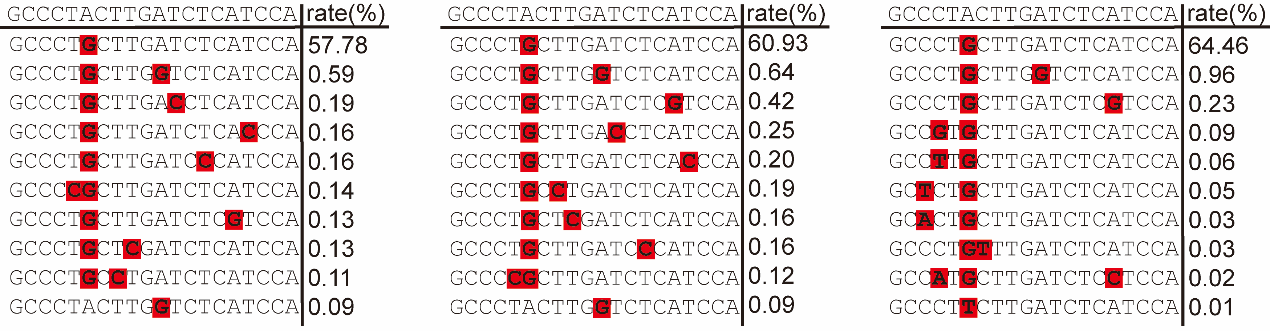


**Supplemental Figure 5.** Specific alleles with base substitution in each PH1 rats treated with 1.0 mg/kg LNP-mRNA at 2-month-old age.


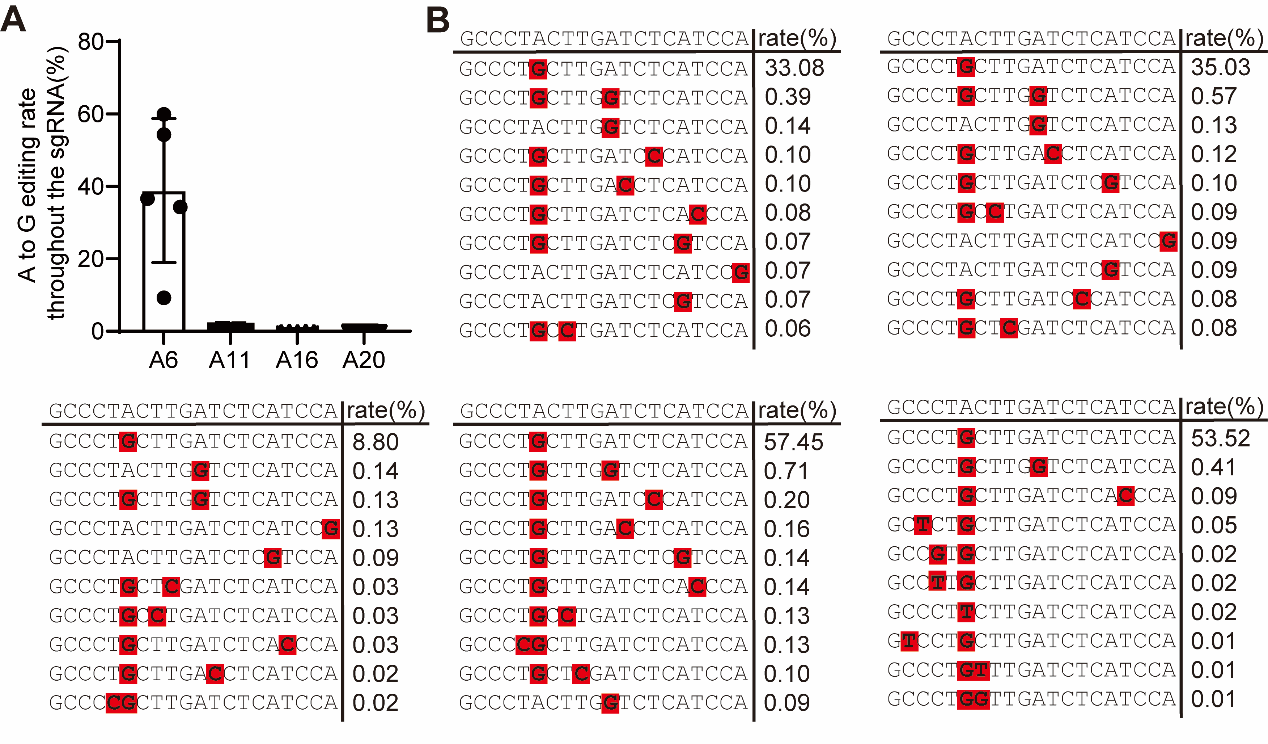


**Supplemental Figure 6.** (A) Assessment of DNA editing efficiency of all adenine (A) sites within the sgRNA *in vivo* 7 days following tail vein injection of 0.35 mg/kg LNP-ABE. (B) Specific alleles with base substitution in each PH1 rat treated with 0.35 mg/kg LNP-ABE treated. Only the top 10 alleles are listed.


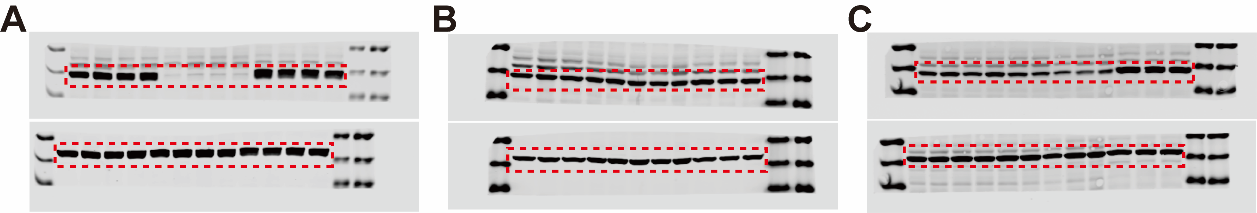


**Supplemental Figure 7.** Uncropped blots of (A) Fig. 2E and (B, C) Fig. 8B.

**Supplemental Table 1.** In silico prediction of the effect of the main bystander mutation on the AGT protein

| Variant | PROVEN score | Prediction (cutoff = -2.5) |
| --- | --- | --- |
| I182T | -4.204 | Deleterious |

**Supplemental Table 2.** List of top 20 predicated off-target sites

| Target ID. | Sequences | PAM | Chromosome | Direction | Position | Mismatch |
| --- | --- | --- | --- | --- | --- | --- |
| Q84X-off-1 | GCCACACTTGATCTCTTCCA | AGA | chr1 | + | 106412476 | 3 |
| Q84X-off-2 | GCCATCCTTGATCTCATCCT | CGA | chr1 | - | 205912434 | 3 |
| Q84X-off-3 | GCCCTACCTGATATCATCAA | TGA | chr2 | + | 135804763 | 3 |
| Q84X-off-4 | GCCATACTTCATCTCATACA | GGA | chr2 | - | 250730762 | 3 |
| Q84X-off-5 | GCCCAACTCCATCTCATCCA | TGA | chr2 | + | 252947167 | 3 |
| Q84X-off-6 | TCCCTACTTGATGTTATCCA | TGA | chr3 | + | 32606584 | 3 |
| Q84X-off-7 | GCACTACTTGATCTCATCTC | TGA | chr6 | + | 20984327 | 3 |
| Q84X-off-8 | GCCCTACCTAATCACATCCA | GGA | chr12 | - | 39832374 | 3 |
| Q84X-off-9 | GCCCTACTTGTTCTCTTCTA | TGA | chr14 | + | 81832554 | 3 |
| Q84X-off-10 | GCCCCAAATGTTCTCATCCA | TGA | chr1 | - | 60605008 | 4 |
| Q84X-off-11 | GACCTACAGGTTCTCATCCA | TGA | chr1 | - | 86093857 | 4 |
| Q84X-off-12 | GCCCGACAGGATGTCATCCA | GGA | chr3 | - | 86693380 | 4 |
| Q84X-off-13 | GCCCTGGATGATGTCATCCA | TGA | chr4 | - | 174583024 | 4 |
| Q84X-off-14 | GCTCTAGTAAATCTCATCCA | AGA | chr13 | - | 56232237 | 4 |
| Q84X-off-15 | GCCAGACTTGAGATCATCCA | AGA | chr14 | + | 73369293 | 4 |
| Q84X-off-16 | ACCCTATCTGATATCATCCA | AGA | chr16 | + | 50058646 | 4 |
| Q84X-off-17 | GTTCTACTTGATCTCATGCT | GGA | chr16 | + | 24498177 | 4 |
| Q84X-off-18 | GACCAACTTAAACTCATCCA | GGA | chr18 | - | 26657913 | 4 |
| Q84X-off-19 | GCTCCACCTAATCTCATCCA | GGA | chr19 | - | 1756940 | 4 |
| Q84X-off-20 | GCTCTGTTAGATCTCATCCA | TGA | chrX | - | 127793096 | 4 |

**Supplemental Table 3.** 24h urinary oxalate of PH1 rats at different times after treatment.

| Collection time | Urinary oxalate μmol/24h | | | | | |  |
| --- | --- | --- | --- | --- | --- | --- | --- |
|  | PH1-Control | WT-Control | PH1-Corrected | PH1-0.25mpk | PH1-0.35mpk | PH1-0.5mpk | PH1-Corrected (Adult) |
| 1 month | 9.1 | 6.2 | 4.2 | N.d. | N.d. | N.d. | 11.8 |
|  | 9.0 | 3.6 | 6.5 | N.d. | N.d. | N.d. | 11.8 |
|  | 7.5 | 3.9 | 3.3 | N.d. | N.d. | N.d. | 9.5 |
|  | 9.2 | 4.2 | 3.2 | N.d. | N.d. | N.d. | / |
|  | 7.6 | 4.9 | 4.1 | N.d. | N.d. | N.d. | / |
|  |  |  |  |  |  |  |  |
| 2 months | 37.8 | 8.3 | 5.8 | 12.0 | 5.5 | 8.7 | 36.0 |
|  | 31.9 | 9.2 | 6.0 | 12.8 | 10.7 | 11.9 | 44.9 |
|  | 29.1 | 9.2 | 6.0 | 11.2 | 12.5 | 6.8 | 44.7 |
|  | 38.3 | 10.3 | 5.5 | 11.0 | 17.3 | 11.3 | / |
|  | 35.8 | 9.2 | 8.8 | 12.0 | 8.6 | 10.7 | / |
|  |  |  |  |  |  |  |  |
| 3 months | 32.0 | 8.9 | 8.9 | 19.2 | 6.0 | 6.7 | 6.0 |
|  | 27.7 | 8.0 | 11.6 | 16.1 | 9.7 | 11.2 | 6.9 |
|  | 29.9 | 10.6 | 7.5 | 12.1 | 9.9 | 7.8 | 7.3 |
|  | 28.8 | 8.2 | 6.1 | 7.5 | 14.2 | 9.2 | / |
|  | 28.3 | 7.8 | 7.3 | 15.0 | 7.2 | 7.5 | / |
|  |  |  |  |  |  |  |  |
| 4 months | 46.8 | 8.1 | 7.5 | N.d. | N.d. | N.d. | 10.5 |
|  | 25.6 | 8.8 | 8.9 | N.d. | N.d. | N.d. | 10.0 |
|  | 26.9 | 8.3 | 8.9 | N.d. | N.d. | N.d. | 8.9 |
|  | 30.9 | 8.3 | 10.8 | N.d. | N.d. | N.d. | / |
|  | 28.6 | 10.5 | 10.5 | N.d. | N.d. | N.d. | / |
|  |  |  |  |  |  |  |  |
| 5 months | 38.4 | 11.7 | 5.7 | N.d. | N.d. | N.d. | 5.6 |
|  | 39.7 | 9.4 | 5.9 | N.d. | N.d. | N.d. | 4.5 |
|  | 37.7 | 8.9 | 5.8 | N.d. | N.d. | N.d. | 4.5 |
|  | 41.3 | 7.5 | 6.0 | N.d. | N.d. | N.d. | / |
|  | 47.6 | 7.9 | 10.3 | N.d. | N.d. | N.d. | / |
|  |  |  |  |  |  |  |  |
| 6 months | 33.3 | 8.4 | 6.3 | N.d. | N.d. | N.d. | 8.6 |
|  | 30.1 | 7.8 | 7.9 | N.d. | N.d. | N.d. | 5.9 |
|  | 29.4 | 6.3 | 6.7 | N.d. | N.d. | N.d. | 8.0 |
|  | 32.4 | 7.3 | 8.1 | N.d. | N.d. | N.d. | / |
|  | 36.5 | 6.3 | 6.1 | N.d. | N.d. | N.d. | / |
|  |  |  |  |  |  |  |  |
| After EG challenge | 114.7 | 19.8 | 69.7 | N.d. | N.d. | N.d. | N.d. |
|  | 100.1 | 84.4 | 55.1 | N.d. | N.d. | N.d. | N.d. |
|  | 117.1 | 26.4 | 21.6 | N.d. | N.d. | N.d. | N.d. |
|  | 109.9 | 69.9 | 45.4 | N.d. | N.d. | N.d. | / |
|  | 113.2 | 45.9 | 13.6 | N.d. | N.d. | N.d. | / |
|  |  |  |  |  |  |  |  |

**Supplemental Table 4.** List of RT-qPCR primers used in this study

| Primer | Sequences |
| --- | --- |
| Rat-q-GAPDH-F3 | gggctcatgaccacagtcca |
| Rat-q-GAPDH-R3 | gtcagatccacaacggatacattgg |
| Rat-Agxt-qPCR-F1 | cctaagaggctcctgttgggt |
| Rat-Agxt-qPCR-R1 | cctgagccgctgacaacc |
| Rat-IL6-qPCR-F | gctctggtcttctggagttccg |
| Rat-IL6-qPCR-R | gatggtcttggtccttagccact |
| Rat-IL10-qPCR-F | gcaggactttaagggttacttggg |
| Rat-IL10-qPCR-R | cattcttcacctgctccactgc |
| Rat-CD68-qPCR-F | tgttcagctccaagcccaaat |
| Rat-CD68-qPCR-R | gctctgatgtcggtcctgtttga |
| qPCR-IL-1β-F | tagcagctttcgacagtgagg |
| qPCR-IL-1β-R | ctccacgggcaagacatagg |

**Supplemental Table 5.** List of NGS primers used in this study

| Primer | Sequences |
| --- | --- |
| Hitom-Q84X-rat genome-F | ggagtgagtacggtgtgcggtggtgatcactgggtagc |
| Hitom-Q84X-rat genome-R | gagttggatgctggatgggttgaacagggcagtctcca |
| hitom-Q84X-OT1-F | ggagtgagtacggtgtgcccatctcaagattcttggtggtc |
| Hitom-Q84-OT1-R | gagttggatgctggatggggccaaatataacagaagagctttcc |
| hitom-Q84X-OT2-F | ggagtgagtacggtgtgctaggcctcctgacccactg |
| hitom-Q84X-OT2-R | gagttggatgctggatggacctcaactggttgtctggc |
| hitom-Q84X-OT3-F | ggagtgagtacggtgtgcgggtgtagttttattgccatggtc |
| hitom-Q84X-OT3-R | gagttggatgctggatgggtctgtctcacctggctatgtattctc |
| hitom-Q84X-OT4-F | ggagtgagtacggtgtgcctgtgccacttcatgatgctg |
| hitom-Q84X-OT4-R | gagttggatgctggatggttcaatgggatcgtgagtttgc |
| hitom-Q84X-OT5-F | ggagtgagtacggtgtgcccaagggcctcacgtcag |
| hitom-Q84X-OT5-R | gagttggatgctggatgggggctgctcaggttgttcaag |
| hitom-Q84X-OT6-F | ggagtgagtacggtgtgcgggggtggatgcttcagaatc |
| hitom-Q84X-OT6-R | gagttggatgctggatggccttgccctctggaatattttctctc |
| hitom-Q84X-OT7-F | ggagtgagtacggtgtgccagacacacacacccctatactca |
| hitom-Q84X-OT7-R | gagttggatgctggatggggggacaggagaccaggt |
| hitom-Q84X-OT8-F | ggagtgagtacggtgtgcgaacaaacgaacaaaggcaaacat |
| hitom-Q84X-OT8-R | gagttggatgctggatgggaaccatccgtaatgagatccgg |
| hitom-Q84X-OT9-F | ggagtgagtacggtgtgcacatgcagtggtttgttcaagagg |
| hitom-Q84X-OT9-R | gagttggatgctggatggcaagctcatcctcatagttatgactacc |
| Hitom-Q84-OT10-F | ggagtgagtacggtgtgcgttcctagcttgggtcccaatca |
| hitom-Q84-OT10-R | gagttggatgctggatggccagtactaggccttacttttagactacc |
| Hitom-Q84-OT11-F | ggagtgagtacggtgtgccaaggtcccacataccagtttca |
| Hitom-Q84-OT11-R | gagttggatgctggatgggccctactcatgggcctcc |
| Hitom-Q84-OT12-F | ggagtgagtacggtgtgcccaggacctagagctagcagc |
| Hitom-Q84-OT12-R | gagttggatgctggatggaacatgaaaaaggtgcgccg |
| Hitom-Q84-OT13-F | ggagtgagtacggtgtgccactgctactgccattactgctt |
| Hitom-Q84-OT13-R | gagttggatgctggatgggttggtggaccctgagttgga |
| Hitom-Q84-OT14-F | ggagtgagtacggtgtgcaaggctgggctcacccaaaa |
| Hitom-Q84-OT14-R | gagttggatgctggatggtgcacaggcaaaaatctcattgct |
| Hitom-Q84-OT15-F | ggagtgagtacggtgtgcctttattattgaaccacaaatgggaccc |
| Hitom-Q84-OT15-R | gagttggatgctggatgggccactctgagcatacattaccaattc |
| Hitom-Q84-OT16-F | ggagtgagtacggtgtgcgccttagcggtagtccacat |
| Hitom-Q84-OT16-R | gagttggatgctggatgggtgtgtaaataaattccatgtttaagcaatggc |
| Hitom-Q84-OT17-F | ggagtgagtacggtgtgcgctccaaaccctaaatatttgcactaacc |
| Hitom-Q84-OT17-R | gagttggatgctggatgggtctgaagggtttctcaaccgga |
| Hitom-Q84-OT18-F | ggagtgagtacggtgtgcagaaaagtagattaggggttccctcc |
| Hitom-Q84-OT18-R | gagttggatgctggatgggcacagccctgccaagtag |
| Hitom-Q84-OT19-F | ggagtgagtacggtgtgccccagaatttcaacacatcatggacttg |
| Hitom-Q84-OT19-R | gagttggatgctggatggtctgggtcttgtatgttggaagtgg |
| Hitom-Q84-OT20-F | ggagtgagtacggtgtgcgcaatatgtttcagtgttttacccagc |
| Hitom-Q84-OT20-R | gagttggatgctggatgggtaggaagtcaacttactgttctgcc |
